# Inverse Design of Active‐Source Metamaterials for Thermal Camouflage with Arbitrary Active Sources

**DOI:** 10.1002/advs.202503024

**Published:** 2025-04-15

**Authors:** Xianrong Cao, Zifeng Tong, Yongle Nian, Jiachang Li, Yinuo Zhou, Zihao Zhang, Yixin Liu, Lei Gong, Zhengdong Cheng, Liqun He

**Affiliations:** ^1^ Department of Thermal Science and Energy Engineering University of Science and Technology of China Hefei 230027 China; ^2^ Department of Optics and Optical Engineering University of Science and Technology of China Hefei 230026 China; ^3^ College of Chemical and Biological Engineering Zhejiang University Hangzhou 310058 China

**Keywords:** active source, active‐source metamaterials, thermal camouflage

## Abstract

Precise control of active‐source thermal fields is critical for advanced technological applications, including thermal camouflage, thermal protection, and energy harvesting. However, the inherent heat generation from active sources often results in localized high‐temperature regions and complex, non‐linear heat flux distributions, posing significant challenges for effectively managing these thermal fields. Here, a novel theoretical design framework is presented for active‐source metamaterials (ASM) by integrating inverse design principles with advanced transformation thermotics. This ASM framework allows for the conversion of complex active‐source effects into tailored anisotropic thermal conductivity distributions, thus enabling precise modulation of active‐source thermal fields for a variety of advanced applications. As a proof‐of‐concept, the precise thermal camouflage of active sources is demonstrated, ranging from simple circular geometries to more complex multi‐leaf configurations, and systematically investigate the interactions between the temperature fields, heat flux distributions, and the power of active sources. Both numerical simulations and experimental validations are conducted to substantiate the effectiveness of the proposed approach. The work establishes a versatile framework for the precise management of active‐source thermal fields, offering significant potential for applications in fields such as chip design, battery technology, and energy systems.

## Graphical abstract

Active‐source metamaterials (ASM) revolutionize thermal camouflage by enabling the precise manipulation of heat signatures, transforming any active source into a desired thermal pattern. This breakthrough technology holds immense potential for infrared stealth, advanced thermal management, and energy‐efficient architecture, offering unprecedented control over heat distribution in diverse applications, from military defense to next‐generation electronics.

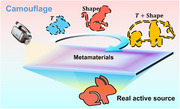

## 1 Introduction

With the rapid advancement of high‐tech fields such as microelectronics, energy systems, and thermal camouflage, the limitations of traditional passive thermal management strategies have become increasingly evident.^[^ ^1^, ^2^, ^3^ ^]^ These approaches, which primarily rely on isotropic heat conduction and natural dissipation, are fundamentally inadequate for meeting the growing demand for precise and adaptive thermal control in modern technologies.^[^ ^4^, ^5^ ^]^ In particular, internal active sources within microchips, batteries, and thermal camouflage devices induce highly localized high‐temperature regions and complex, non‐uniform heat flux distributions, which not only degrade system performance and efficiency but also pose severe reliability risks, including thermal runaway and material fatigue.^[^ ^6^, ^7^, ^8^, ^9^ ^]^ Moreover, in applications requiring thermal camouflage and stealth, precise modulation of temperature distributions is essential for concealing thermal signatures and achieving robust thermal protection.^[^ ^10^, ^11^ ^]^ Addressing these challenges necessitates the development of innovative strategies capable of actively managing active‐source thermal fields, thereby unlocking new frontiers in energy systems, functional thermal materials, and next‐generation thermal management systems.^[^ ^12^, ^13^, ^14^ ^]^

In recent years, thermal metamaterials have emerged as a promising solution to overcome the limitations of traditional passive thermal management systems.^[^ ^15^, ^16^, ^17^, ^18^, ^19^ ^]^ These advanced materials enable the precise manipulation of heat flux through engineered anisotropic thermal conductivities, which can be tailored to meet specific thermal needs across a wide range of applications. Notable devices, such as thermal cloaks that conceal heat signatures,^[^ ^20^, ^21^, ^22^, ^23^ ^]^ thermal lenses that redistribute heat,^[^ ^24^ ^]^ and thermal diodes that facilitate unidirectional heat transport,^[^ ^25^, ^26^ ^]^ have demonstrated the transformative potential of these metamaterials, enabling advanced thermal control in complex and high‐performance systems. However, most existing thermal metamaterial designs remain passive, typically treating active sources as either nonexistent or simplified to static high‐temperature boundary conditions.^[^ ^27^, ^28^ ^]^ Such assumptions are inadequate in real‐world thermal systems, where active sources with specific power outputs generate localized, high‐temperature regions, resulting in nonlinear heat flux distributions. The nonlinear nature of these active‐source thermal fields goes beyond the capabilities of traditional transformation thermotics, which focus on thermal properties like conductivity and specific heat.^[^ ^6^, ^29^, ^30^ ^]^ Furthermore, unlike thermal conductivity—an inherently vectorial property that can be manipulated within existing transformation frameworks—active sources, being scalar quantities, present unique challenges that cannot be captured through simple vectorial transformations. To address these issues, an active thermal management approach is required, one that enables adjustments of material properties in response to the power outputs of active sources. By integrating active‐source management into the framework of thermal metamaterials, this new paradigm offers the potential for unprecedented levels of thermal control, thereby opening up new avenues for applications in microelectronics, energy systems, and beyond.

Here, we propose a novel design framework for ASM. By integrating inverse design principles and advanced transformation thermotics,^[^ ^31^, ^32^ ^]^ we establish a unified approach to tailor the thermal conductivity distribution under active‐source conditions. This method allows us to translate the complex impact of active sources on the thermal fields into changes in the thermal conductivity distribution, offering precise control over temperature fields and heat flux distributions. We demonstrate the potential of this framework through a variety of thermal camouflage devices, where real active sources can take on arbitrary shapes and power levels. Interestingly, once the thermal camouflage design is implemented, both the shape and temperature of the active sources can be precisely controlled. Numerical simulations and experimental validations reveal unprecedented control over complex thermal fields, paving the way for robust solutions in real‐world applications. This work not only provides a general design framework for ASM design in the presence of active sources, but also may extend to the study of active‐source systems in optics, acoustics, mechanics, and other fields.

## 2 Results and Discussion

### 2.1 The Design Theoretical Framework of ASM

**Figure 1:**
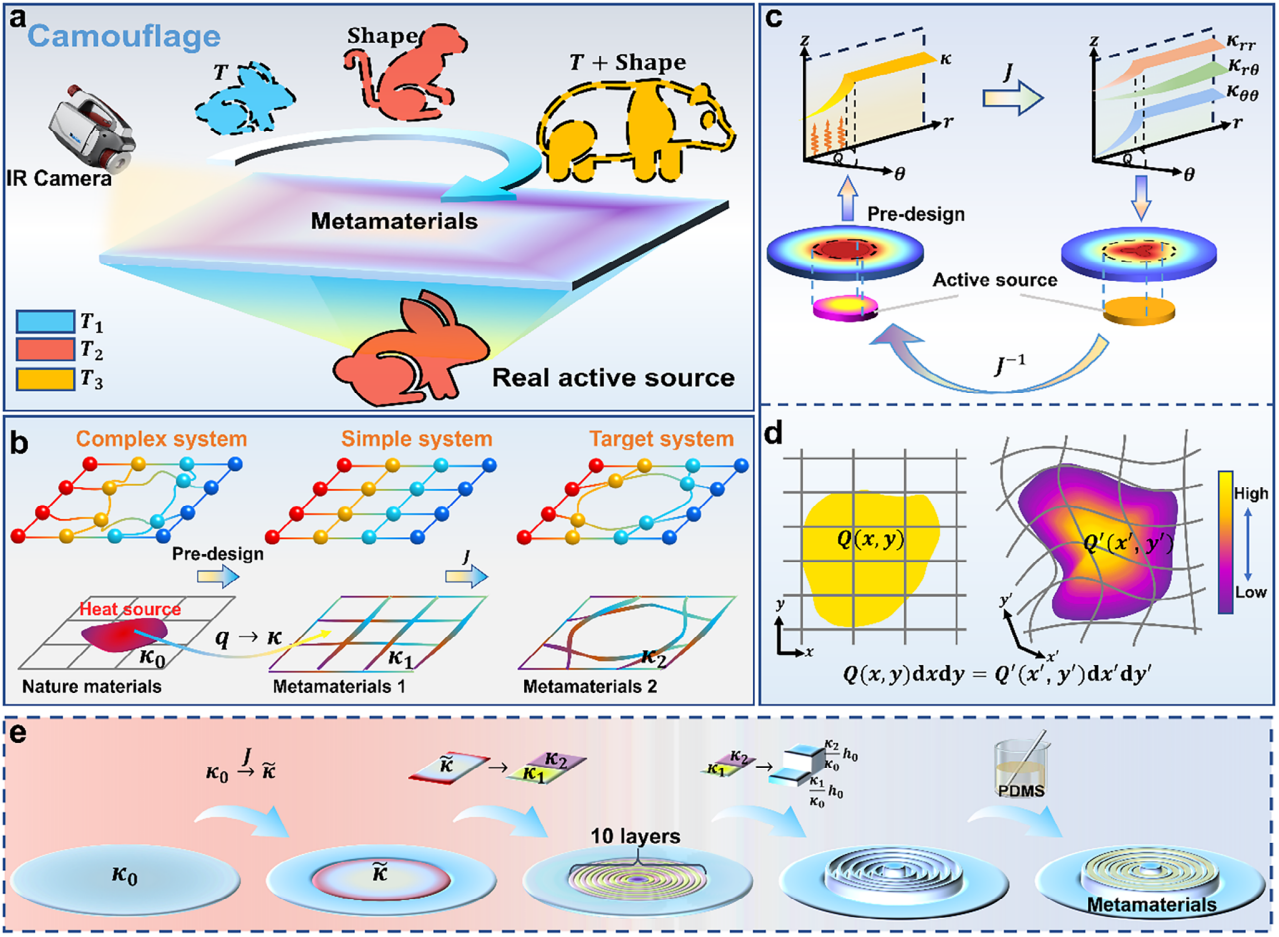
Schematic and design principles of ASM. a) Schematic of ASM for thermal camouflage, enabling independent disguise of both the shape and temperature of a rabbit‐shaped active source. b) Design principle of ASM, where the impact of the active source on the thermal field is translated into modifications of material properties, thereby achieving functional regulation of active‐source thermal fields. c) Demonstration of the design process, using an example where a circular active source is camouflaged as a clover‐shaped thermal signature. d) Theoretical framework of active source transformation. As a scalar quantity, the active source *Q* satisfies the principle of energy conservation, ensuring that the power per unit area remains invariant in both the original and transformed domains. e Flowchart of the ASM design process.

The design of ASM introduces fundamental challenges beyond conventional passive systems, as active sources inherently reshape the thermal fields, generating nonlinear temperature fields and complex heat flux distributions.^[^ ^33^, ^34^, ^35^ ^]^ To address this, we establish an inverse design framework that reformulates active‐source‐induced thermal field control into a mathematically tractable problem (**Figure** **1**b). Instead of directly solving for the complex thermal field, we first map it onto a simplified active‐source thermal field where active‐source effects can be systematically analyzed (Figure 1c). By determining the thermal conductivity distribution in this transformed space, our approach enables precise control of active‐source thermal fields under arbitrary active‐source configurations, laying the foundation for next‐generation thermal metamaterials with unprecedented control capabilities.

Next, we present the theoretical framework for the inverse design methodology in 2D polar coordinates. The transformation relationship between the target thermal field and the preset simple thermal field is established as follows:

(1) $$\left\{\begin{array}{c} \;r^{\prime }=r^{\prime }\;\left(r,\theta \right)\\ \;\theta^{\prime }=\theta^{\prime }\;\left(r,\theta \right) \end{array}\right.$$

where *r*′ and θ′ represent the radial distance and polar angle in the target thermal field, respectively, while *r* and θ correspond to those in the preset simple thermal field. According to the principles of transformation thermotics,^[^ ^36^ ^]^ the associated coordinate transformation matrix is given by:

(2) $$J=\left(\begin{array}{cc} 1 & 0\\ 0 & r^{\prime } \end{array}\right)\;\left(\begin{array}{cc} \frac{\partial r^{\prime }}{\partial r} & \frac{\partial r^{\prime }}{\partial \theta }\\ \frac{\partial \theta^{\prime }}{\partial r} & \frac{\partial \theta^{\prime }}{\partial \theta } \end{array}\right)\left(\begin{array}{cc} 1 & 0\\ 0 & \frac{1}{r} \end{array}\right)$$

Next, we focus on determining the distribution of the target active source within the preset simple thermal field. It is crucial to emphasize that, unlike traditional transformation thermotics, the active source cannot be directly manipulated through coordinate transformation. This limitation arises due to the fact that active sources, unlike other thermodynamic quantities, cannot be reduced to simple vectorial transformation. Consequently, traditional methods fail when attempting to manage thermal fields affected by the presence of active sources.

To address this, we turn to the law of energy conservation, which mandates that the total power of the active source remains invariant before and after the transformation (Figure 1d). This principle allows us to derive the following energy balance equation:

(3) $$\int \;\;\;\int_{D}q(r,\theta )dS=\int \;\;\;\int_{D^{\prime }}q^{\prime }\left(r^{\prime },\theta^{\prime }\right)dS^{\prime }$$

where *q* and *q*′ represent the active source power distribution functions in the preset simple thermal field and the target thermal field, respectively, while *D* and *D*′ denote the active source regions in the original and transformed coordinates. The differential elements d*S* and d*S*′ correspond to the areas in these regions. With the active source distribution *q*′ known in the target thermal field, we can integrate the equation using the coordinate transformation relationship to solve for *q* in the preset thermal field. Upon determining the active source power in the preset thermal field, we proceed to solve the steady‐state Fourier heat conduction equation:

(4) $$\nabla \left(\kappa \nabla T\right)+q=0$$

where *T* and *κ* represent the temperature field and thermal conductivity, respectively, and *q* denotes the active‐source power distribution. Assuming the isotropic thermal conductivity, we expand the equation in polar coordinates for two dimensions:

(5) $$\frac{1}{r}\frac{\partial \left(\kappa r\frac{\partial T}{\partial r}\right)}{\partial r}+\frac{1}{r^{2}}\frac{\partial \left(\kappa \frac{\partial T}{\partial \theta }\right)}{\partial \theta }+q=0$$

For computational efficiency, we introduce substitution variables ε = φ(*r*,  θ) and ω = ϕ(*r*,  θ), rewriting the equation in a general form:

(6) $$\begin{array}{ccc} \left(rf_{1}\frac{\partial \varphi }{\partial r}+\frac{1}{r}f_{2}\frac{\partial \varphi }{\partial \theta }\right)\frac{\partial \kappa }{\partial \varepsilon } & + & \left(rf_{1}\frac{\partial \phi }{\partial r}+\frac{1}{r}f_{2}\frac{\partial \phi }{\partial \theta }\right)\frac{\partial \kappa }{\partial \omega }+\\ & & \left(f_{1}+rf_{3}+\frac{f_{4}}{r}\right)\kappa +rq=0 \end{array}$$

Here, *f*_1_ = ∂*T*/∂*r* , *f*_2_ = ∂*T*/∂θ , *f*_3_ = ∂^2^ *T*/∂*r* ^2^ , and *f*_4_ = ∂^2^ *T*/∂θ^2^ , representing the first and second partial derivatives of the temperature field. Since the preset thermal field is relatively simple, solving for the first and second derivatives of the temperature field is straightforward. To simplify the analysis, we take ω = ϕ(*r*,  θ), ensuring that it satisfies the following equation:

(7) $$rf_{1}\frac{\partial \phi }{\partial r}+\frac{1}{r}f_{2}\frac{\partial \phi }{\partial \theta }=0$$

Thus, Equation (6) reduces to a first‐order partial differential equation with respect to ε. Then, by solving Equation 7, we obtain the explicit function for ω. Utilizing the Jacobian matrix, we find an ε function such that:

(8) $$J\left(\varepsilon ,\omega \right)=\frac{\partial \left(\varphi ,\phi \right)}{\partial \left(r,\theta \right)}\neq 0$$

Substituting ε and ω into Equation (6), we can obtain:

(9) $$\frac{\partial \kappa }{\partial \varepsilon }+\frac{f_{1}+rf_{3}+\frac{f_{4}}{r}}{rf_{1}\frac{\partial \varphi }{\partial r}+\frac{1}{r}f_{2}\frac{\partial \varphi }{\partial \theta }}\kappa +\frac{rq}{rf_{1}\frac{\partial \varphi }{\partial r}+\frac{1}{r}f_{2}\frac{\partial \varphi }{\partial \theta }}=0$$

The prescribed thermal conductivity distribution within the preset thermal field can ultimately be obtained by solving Equation (9):

(10) $$\kappa =\frac{\int \nu e^{\int \mu d\omega }d\omega +f\left(\varepsilon \right)}{e^{\int \mu d\omega }}$$

whereμ  = (*f*_1_ + *rf*_3_ + *f*_4_/*r*)/(*rf*_1_∂ϕ/∂*r* + *f*_2_/*r*∂ϕ/∂θ) and ν  = *rq*/(*rf*_1_∂φ/∂*r* + *f*_2_/*r*∂φ/∂θ) . *f*(ε) is a function of ε. By solving this, we obtain the thermal conductivity distribution within the preset thermal field. Subsequently, the thermal conductivity distribution within the target functional region can be derived based on the coordinate transformation relations.

(11) $$\kappa^{\prime }=\frac{J\kappa J^{T}}{\det\left(J\right)}$$

κ′ represents the thermal conductivity in the target thermal field. Our design process is general and can be readily extended to 3D domains (see Supplementary Material S1 for detailed derivations).

### 2.2 ASM‐Based Shape Camouflage for Active Sources with Precise Thermal Management

Thermal camouflage, as a pivotal application of active‐source thermal field management, has attracted significant research interest over the years.^[^ ^37^, ^38^ ^]^ However, conventional thermal camouflage strategies often lack the capability to precisely control both the temperature and shape of the camouflaged active source, thereby limiting their applicability in complex thermal environments.^[^ ^7^, ^39^, ^40^, ^41^, ^42^ ^]^ Here, we propose an advanced approach based on ASM that enables simultaneous shape transformation and temperature regulation (Figure 1a). Leveraging the unique properties of these metamaterials, our method achieves independent and highly precise control over the thermal signature, allowing for the advanced camouflage of active sources with diverse geometries, ranging from simple shapes to intricate multi‐leaf configurations. This unprecedented level of thermal control is not only essential for enhancing the effectiveness of thermal camouflage but also establishes a foundation for more sophisticated strategies in active‐source thermal management.

**Figure 2:**
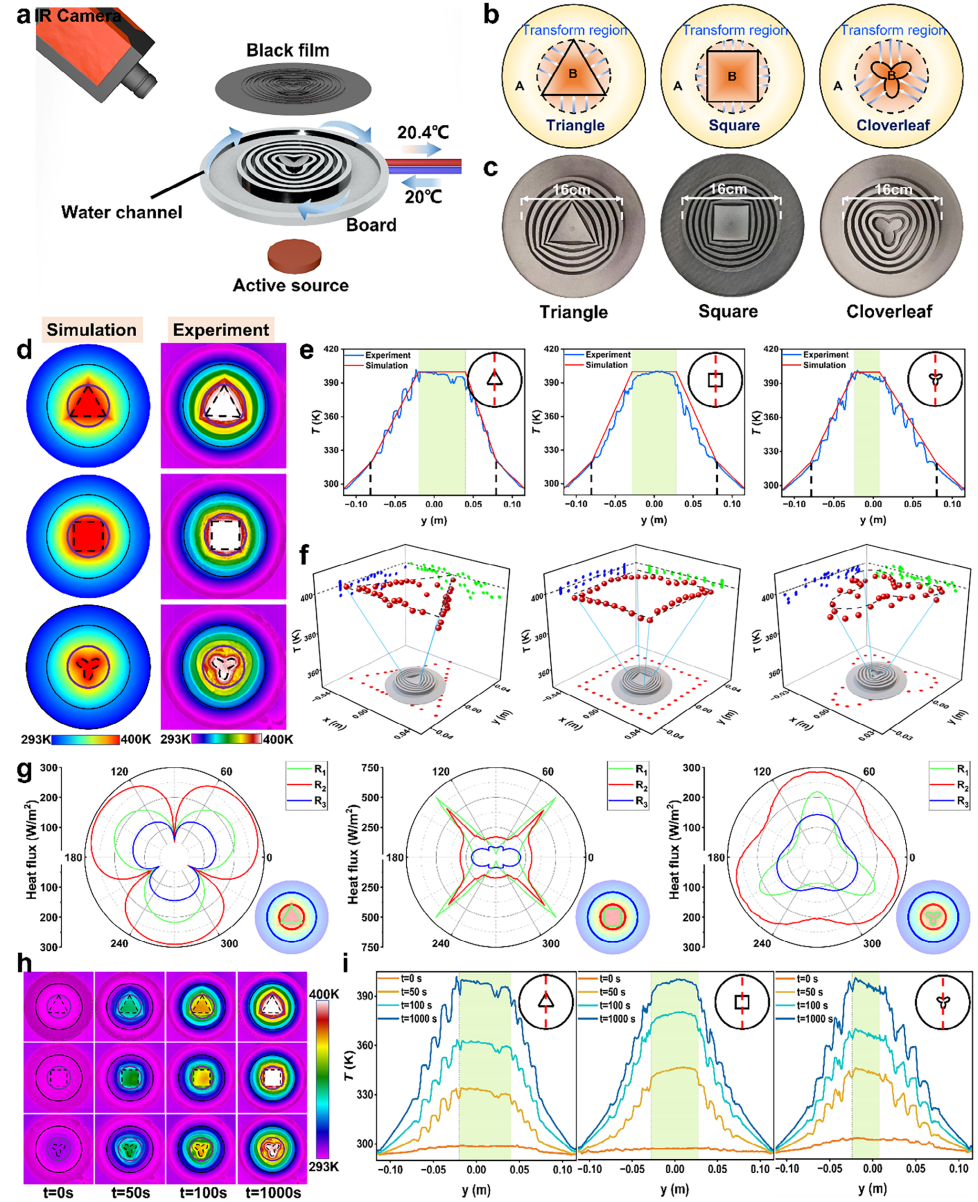
Experimental and simulated analysis of shape transformation in active‐source thermal camouflage. a) Experimental setup. b) Schematic of spatial transformations camouflaging the circular active source into triangular, square, and clover‐shaped thermal signatures. c) Top view of the experimental plates. d) Temperature heatmap of simulation and experimental results. e) Temperature profiles along the vertical central axis in both simulation and experimental results. f) Temperature analysis at the boundary of the camouflaged active source, where red spheres indicate experimental measurements. The blue and green points in this figure represent the projections of the experimental data (red points) onto the YZ and XZ planes, respectively. g) Heat flux analysis at the boundaries of the camouflaged active source, the actual active source, and the designed transformation region. h) Transient experimental results. i) Temperature profiles along the vertical central axis.

To rigorously validate the capabilities of ASM, we design and simulate three thermal camouflage schemes for the most fundamental and widely encountered case—a circular active source (see Supplementary Material S2 for details). By applying a radial coordinate transformation, the circular active source reshapes into triangular, square, and clover‐shaped thermal profiles while maintaining an undisturbed external thermal field (**Figure** **2**b). To achieve this, we establish a preconfigured thermal environment with a maximum central temperature of 400 K, and heat flux flows radially outward to ensure that the transformation does not disrupt the inherent heat flux characteristics. Finite element simulations are conducted using COMSOL Multiphysics 6.3, with structural parameters set as *R*_1_ =  0.04 m, *R*_2_ =  0.08 m, and *R*_3_ =  0.12 m, where *R*_1_ represents the radius of the circular active source, heated at 10000 W·m⁻^2^, while *R*_2_ and *R*_3_ define the transformation region and the surrounding background, respectively. The annular background (*R*_2_ − *R*_3_) has a thermal conductivity of 0.25 W·m⁻¹·K⁻¹, and the system's outer boundary remains at 293.15 K. Simulation results confirm that the circular active source successfully camouflages into triangular, square, and clover‐shaped thermal distributions, with the external temperature field remaining unperturbed (Figure 2d, black dashed lines). Further quantitative analysis along the vertical central axis (Figure 2e, red line) demonstrates that the transformed active source consistently maintains the target temperature of 400 K, while the background region (*R*_2_ − *R*_3_) remains thermally stable, and the transformation boundary exhibits a temperature of 323 K. Heat flux analysis at three critical locations—the camouflaged active source boundary, the original active source boundary, and the transformation domain boundary (Figure 2g, green, red, and blue curves, respectively)—reveals that heat flux maximizes at the original circular active source boundary and minimizes at the camouflaged boundary, a direct consequence of radial heat transport, where thermal conduction paths intersecting the active source concentrate energy input, whereas, in regions without direct heating, the increasing conduction area leads to a natural reduction in heat flux density. This conduction‐based thermal camouflage strategy offers a fundamentally new approach to high‐fidelity thermal regulation, ensuring that the active source precisely maintains its designated temperature without disturbing its surroundings. Compared to conventional radiation‐based thermal camouflage, our ASM strategy exhibits superior stability and adaptability, making it a promising candidate for advanced thermal management applications.

To experimentally validate our theoretical framework, we construct a dedicated experimental system (Figure 2a). The camouflaged panel follows the fabrication process depicted in Figure 1e, where its core geometric structure is 3D‐printed using aluminum alloy (κ = 40 W·m⁻¹·K⁻¹) (Figure 2c). The central active source, designed to match the theoretical settings, delivers a power density of 10 000 W·m⁻^2^ over a radius of 0.04 m and is composed of cast aluminum. To establish a well‐defined boundary condition, an annular water channel at the panel's periphery is connected to a thermostatic water bath, maintaining a stable low‐temperature boundary of 293.15 K. To ensure uniform infrared emissivity (*ε *= 1), the panel surface is coated with carbon black, and an infrared thermal imaging camera is employed for real‐time thermal visualization. The experimental results (Figure 2d) confirm that the circular active source is effectively camouflaged into triangular, square, and clover‐shaped thermal distributions, with the external thermal field remaining undisturbed. A quantitative analysis along the vertical central axis (Figure 2e, blue line) exhibits excellent agreement with theoretical predictions within experimental uncertainty, further demonstrating that the designed transformation does not introduce thermal distortions in the background region. Moreover, temperature measurements at the camouflaged active source boundaries indicate that the boundary temperatures remain consistently around 400 K (Figure 2f, red dots), verifying the precision of our metamaterial‐based thermal regulation. To further characterize its dynamic response, transient experiments are conducted at *t* = 0 s, *t* = 50 s, *t* = 100 s, and *t* = 1000 s (Figure 2h), with temperature profiles along the vertical axis revealing the intrinsic transient characteristics of the ASM. These results collectively establish a solid experimental foundation for high‐fidelity conduction‐based thermal camouflage, demonstrating its effectiveness in real‐time thermal field manipulation and laying the groundwork for advanced applications in thermal stealth, microelectronics, and energy management.

### 2.3 ASM‐Based Thermal Camouflage for Active Sources: Shape Transformation and Temperature Regulation

Precise temperature (*T*) control is a fundamental aspect of active‐source thermal field management, particularly in advanced technologies such as microelectronics, batteries, and energy storage systems. In these fields, thermal runaway has become a critical limiting factor, impeding further progress. Existing solutions, which rely on heat transfer mechanisms such as convection and phase change, can mitigate some of these challenges. However, they often fall short of providing the level of precision and efficiency required for effective thermal management of heat‐generating devices. Therefore, there is an urgent need for innovative and highly efficient strategies to regulate device temperature. In this context, the ASM we propose offers a promising solution, providing a more effective approach to temperature regulation in heat‐generating devices.

**Figure 3:**
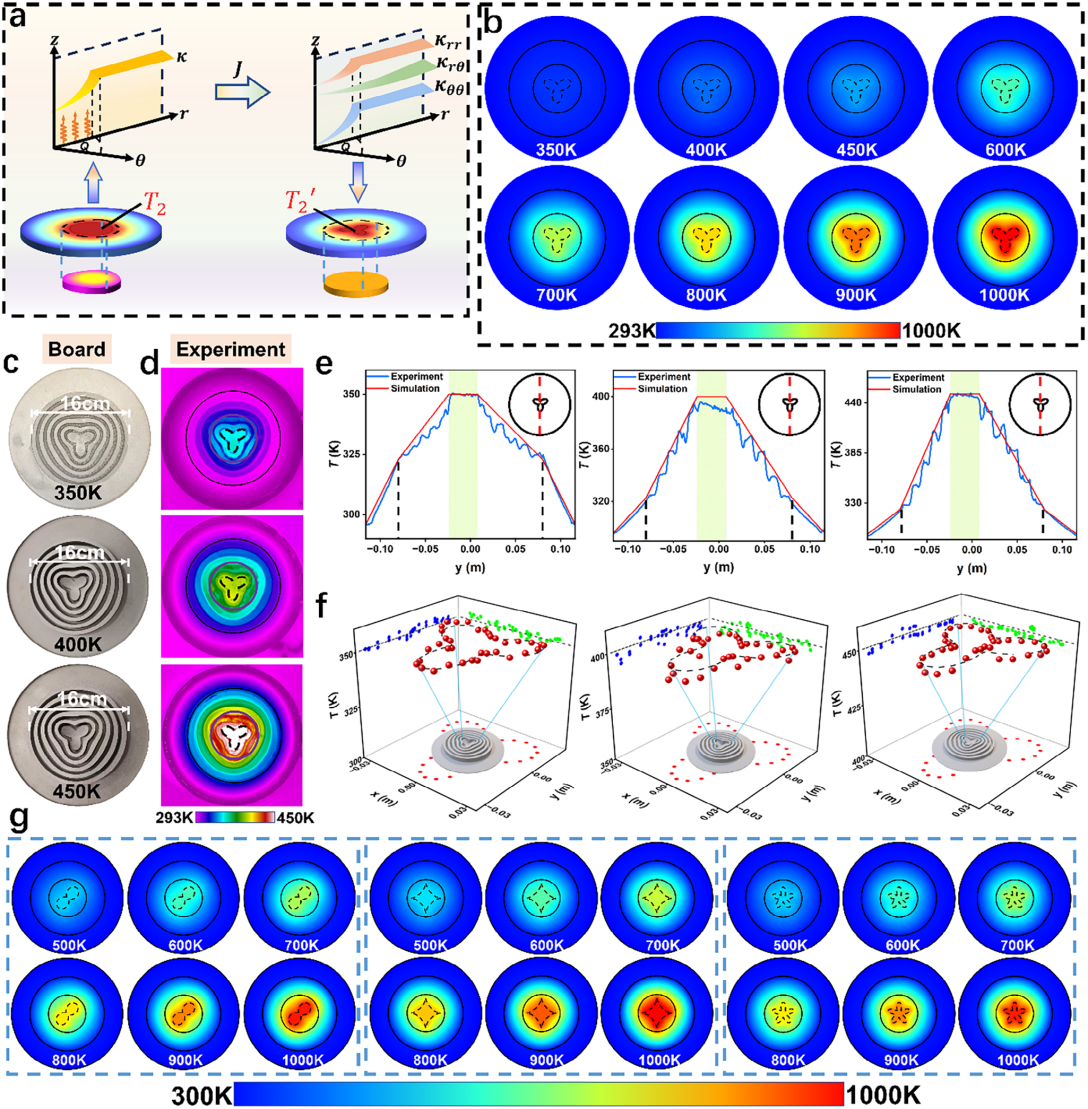
Experimental and simulation analysis of temperature control in active‐source thermal camouflage. a) Schematic diagram illustrating the design for temperature control of camouflaged active sources. b) Temperature heatmap showing the camouflage of the active source into a clover shape, with simultaneous regulation of the active‐source temperature from 350 to 1000 K. c) Top view of the experimental plates. d) Experimental temperature heatmap results. e) Temperature profile comparison between experimental and simulation results along the vertical central axis. f) Temperature analysis at the boundary of the camouflaged clover‐shaped active source. g) Temperature heatmap showing the transformation of a circular active source into two‐leaf clover, four‐point star, and five‐point star shapes, with simultaneous temperature regulation from 500 to 1000 K.

We investigate the use of ASM for precise control of active‐source temperatures. As a conceptual demonstration, we focus on camouflaging a circular active source into a clover shape while simultaneously regulating its temperature (for a detailed design process, refer to Supplementary Material S2). **Figure** **3**a outlines the theoretical framework for this approach, where the temperature *T*_2_ of the active source in a preset thermal field is manipulated. Through a coordinate transformation, the resulting active‐source temperature *T*_2_′ in the target field can be accurately controlled. Using the same simulation parameters as in Figure 2, we obtain the simulation results depicted in Figure 3b. These results show that the circular active source is successfully camouflaged into a clover shape, with the active‐source temperature varying within a range of 350 to 1000K. A temperature profile along the vertical central axis (Figure S6b, Supporting Information) shows that the background thermal field remains undisturbed, and the temperature at the boundary of the transformation region is consistently maintained at 323K. Additionally, an analysis of thermal conductivity reveals that the thermal conductivity in the background region remains unchanged, while in the transformation region, the thermal conductivity components (κ_*xx*_, κ_*xy*_/κ_*yx*_, and κ_*yy*_) decrease as the active‐source temperature increases (Figure S6c, Supporting Information). Subsequently, we employ the same experimental design method and obtain the corresponding experimental plates and results (Figure 3c,d). These experimental results confirm that the circular active source (P = 10 000 W·m⁻^2^) is successfully camouflaged into a clover shape, with the temperatures corresponding to 350, 400, and 450K. Figure 3e shows the temperature distribution along the vertical central axis, where the experimental results closely align with the theoretical predictions, within the expected error margins. Furthermore, temperature measurements at the camouflaged active‐source boundary indicate that the temperatures remain stable at 350, 400, and 450K (Figure 3f). These simulations and experimental results provide compelling evidence of the efficacy of ASM in regulating the temperature of active sources, demonstrating their potential for precise and reliable thermal management.

To further illustrate the versatility of our design theory, we extend the concept of thermal camouflage by transforming the circular active source into various geometries, including a two‐leaf clover, a four‐point star, and a five‐leaf shape (Figure 3g). In the simulations, we set the parameters as *R*_1_ =  0.05 m, *R*_2_ =  0.1 m, *R*_3_ =  0.15 m, with a circular active source (*R*_1_ =  0.05 m) power of 10 000 W·m⁻^2^. Figure S8a (Supporting Information) illustrates the radial heat flux direction. Figure S8b (Supporting Information) presents the temperature distribution along the vertical central axis, where the background region remains thermally stable, while the temperature of the camouflaged active source varies from 500 to 1000K. Additionally, we analyze the heat flux at key boundaries: the camouflaged active source, the original circular active source, and the design region (Figures S7b and S8c, Supporting Information). The results confirm that, despite the transformation to different thermal camouflage shapes and temperatures, the heat flux distribution remains unaffected, thereby demonstrating the robustness of the approach in achieving efficient thermal management.

### 2.4 ASM‐Based Thermal Camouflage for Active Source with Arbitrary Complex Geometries and Power Levels

The management of thermal fields for active sources with arbitrary shapes, sizes, and power levels remains a significant challenge in advanced thermal regulation. Unlike conventional systems that often assume simplified active‐source geometries, real‐world applications, such as microelectronics, batteries, and energy storage devices, involve complex and diverse heat generation patterns. These systems are prone to thermal runaway, where uneven or inefficient heat dissipation can lead to device failure or compromised performance. This limitation underscores the urgent need for a versatile approach capable of managing arbitrary active sources.

**Figure 4:**
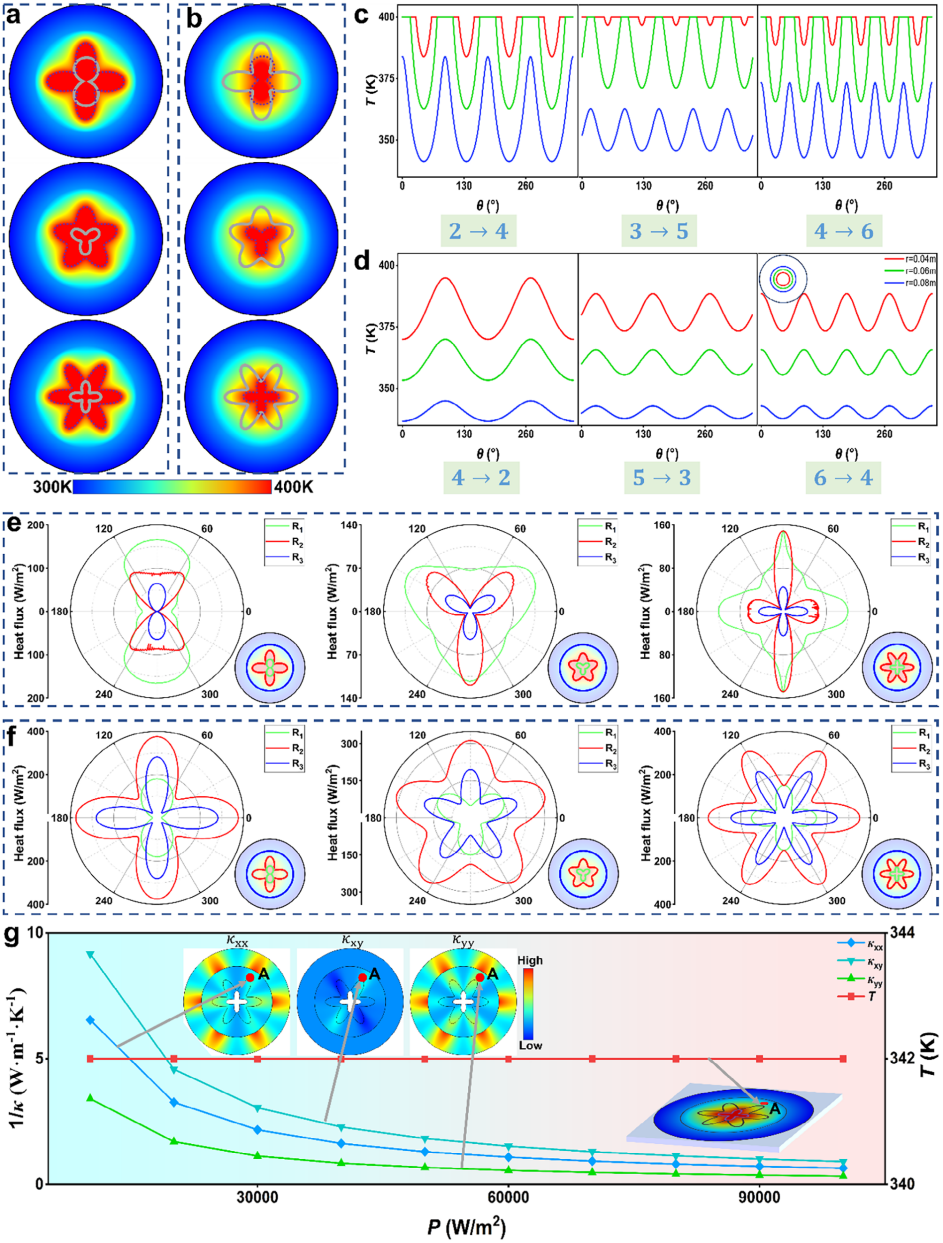
Simulation results and analysis of thermal camouflage for active sources with various shapes and power levels. a) Simulation results demonstrating the camouflage of two‐leaf, three‐leaf, and four‐leaf shapes of active sources into four‐leaf, five‐leaf, and six‐leaf shapes, respectively. The gray solid lines indicate the actual active sources, while the black dashed lines represent the camouflaged active sources. b) Simulation results illustrating the four‐leaf, five‐leaf, and six‐leaf shapes of active sources are camouflaged into two‐leaf, three‐leaf, and four‐leaf shapes, respectively. c and d Temperature profiles along circular paths with radii of 0.04, 0.06, and 0.08 m for the cases shown in a) and b), respectively. e) and f) Heat flux analysis at the boundaries of the actual active source, camouflaged active source, and transformation region. g) Temperature and thermal conductivity variations at point A (marked in case 6 → 4, *r* = 0.08 m and θ = 60°) under different active‐source power levels while maintaining the same camouflage functionality.

To validate this extended capability, we conduct numerical simulations on the camouflage design of active sources with six different geometries, using the same simulation parameters as those presented in Figure 3f. **Figure** **4**a illustrates the transformation of real active sources in the shape of a two‐leaf clover, a three‐leaf clover, and a four‐leaf clover into camouflaged shapes of a four‐leaf clover, a five‐leaf clover, and a six‐leaf clover while maintaining the active‐source temperature at 400K. Notably, the camouflaged active sources (black dashed lines) are observed to be larger than their original counterparts (gray solid lines). Conversely, Figure 4b shows the transformation of four‐leaf clover, five‐leaf clover, and six‐leaf clover‐shaped active sources into two‐leaf clover, three‐leaf clover, and four‐leaf clover shapes, with the camouflaged active sources appearing smaller than the original shapes. Figure 4c,d presents an analysis of the temperature distribution along the radii of *r* = 0.04 m, *r* = 0.06 m, and *r* = 0.08 m, where the number of temperature peaks corresponds directly to the shape of the camouflaged active source. Furthermore, the temperature profile along the vertical central axis, as shown in Figure S11 (Supporting Information), confirms that the active‐source temperature is precisely maintained at 400K, while the background temperature field remains undistorted, thereby demonstrating the fidelity of the transformation. In Figure 4e,f, we present a heat flux analysis at the boundaries of the camouflaged active source, the real active source, and the transformation region. The heat flux distribution shows that the number of heat flux peaks is directly related to the real geometry of the active source, with the larger real active source corresponding to a higher heat flux density.

Additionally, we investigate the relationship between temperature and thermal conductivity in the context of varying active‐source powers while achieving the same thermal camouflage effect. For illustration, we focus on point A from the 6 → 4 camouflage result. As depicted in Figure 4g, when the active‐source power is increased from 10 000 to 100000 W·m⁻^2^, the reciprocal of thermal conductivities (1/κ_*xx*_, 1/κ_*xy*_ and 1/κ_*yy*_) gradually decrease to maintain a constant temperature. Concurrently, the heat flux density increases with the power, as shown in Figure S15 (Supporting Information). These results, demonstrating the successful camouflage of active sources with varying shapes and power levels, substantiate the generality and effectiveness of ASM design theory. This has significant application potential in real‐world thermal camouflage scenarios. For example, it could be used to disguise the infrared thermal signature of a tank as a deer, effectively deceiving the enemy. Moreover, it also provides a novel solution for advanced thermal management in high‐tech applications such as chips, batteries, and energy storage systems.

## 3 Conclusion

In this study, we introduce a novel design framework for ASM, enabling efficient thermal management of complex active‐source thermal fields in an entirely new manner. As a conceptual demonstration, we showcase the capability of these metamaterials to effectively camouflage active sources of arbitrary shape, size, and power. This approach allows for precise manipulation of both the shape and temperature of the active source while ensuring that the surrounding thermal environment remains undisturbed. Additionally, we explore the interplay between active‐source power, anisotropic thermal conductivity, temperature, and heat flux. Our analysis reveals a linear relationship: as the power of the active source increases, both thermal conductivity and heat flux also increase, ensuring that the thermal camouflage functionality remains unaltered. The results from both simulations and experiments consistently validate the robust capability of ASM in regulating and controlling the active‐source thermal fields. This work not only establishes a solid theoretical foundation for the design of ASM but also provides a novel solution for advanced thermal management in applications such as microelectronics, batteries, and thermal camouflage.

## 4 Experimental Section

### Experimental Design

Inspired by the work of *Schittny* et al.^[^ ^43^ ^]^ equivalent anisotropic thermal conductivity could be theoretically achieved by arranging ten alternating layers with high and low thermal conductivity in a radial pattern. The equivalent anisotropic thermal conductivity is given by:

(12) $$\kappa_{x}^{=}f\kappa_{\mathrm{low}}+\left(1-f\right)\kappa_{\mathrm{high}}$$

(13) $$\kappa_{y}={\left(f/\kappa_{\mathrm{low}}+\left(1-f\right)/\kappa_{\mathrm{high}}\right)}^{-1}$$

Here, *f* represents the proportion of low thermal conductivity, while 1 − *f* represents the proportion of high thermal conductivity. κ_low_ and κ_high_ represent the thermal conductivities of low and high materials, respectively. To achieve optimal experimental results, the effects of actual convection were considered during the optimization process, with the convection heat transfer coefficient set to 10 W · m^−2^ · K^−1^. The core of the optimization algorithm was to uniformly select 11 points along the panel's central axis and minimize the standard deviation from theoretical values.

(14)

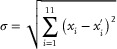




Here, *x_i_* represents the measured temperature values during the optimization process, and $x_{i}^{\prime }$ represents the theoretical values. Additionally, since materials with extremely high thermal conductivity do not exist in nature, fitting the thermal conductivity distribution using higher‐order functions was proposed to achieve a more uniform temperature field in the panel center. This approach resulted in a very small temperature gradient within the camouflaged active‐source region. Simulation tests showed that a parabolic thermal conductivity distribution makes the temperature in the central region very uniform. Finally, by converting the thermal conductivity into the corresponding heights of the thermal channel dimensions, the heights of the experimental panels were obtained, as shown in Table S1 (Supporting Information).

### Numerical Simulations

Steady‐state simulations were conducted using the commercial software COMSOL Multiphysics with the Heat Transfer Module. Each ASM used for camouflaging a circular active source has the same dimensions, with the parameters: *R*_1_ =  0.04 m, *R*_2_ =  0.08 m, and *R*_3_ =  0.12 m (or *R*_1_ =  0.05 m, *R*_2_ =  0.1 m, and *R*_3_ =  0.15 m). It is worth noting that the chosen dimensions were selected for experimental convenience. In practice, the design dimensions can be arbitrary, provided they do not fall below the limits of manufacturing precision. The thermal conductivity of the background region is  0.25 W · m^−2^ · K^−1^, with a boundary temperature of ambient temperature *T*_0_ =  293.15 K. The initial temperature of the entire system was also *T*_0_. Details of the thermal conductivity distribution for various thermal metamaterials were provided in Supplementary Materials S2, Figure S4 (Supporting Information). In addition, to optimize the design of the experimental materials, realistic 3D models were constructed, and conduct simulation analyses, as detailed in Supplementary Materials S4 (Supporting Information). The thermal conductivity was set to be equivalent to that of aluminum alloy (κ_Al_ =  176 W · m^−2^ · K^−1^). To simulate real conditions, natural convection boundary conditions were introduced with a convective heat transfer coefficient *h*  =  10 W · m^−2^ · K^−1^. Moreover, to mitigate the effects of convection, the material structure was optimized using advanced modules. It was found that the final temperature field closely resembled that obtained from simplified 2D simulations. Finally, to camouflage arbitrary active sources, the simulation parameters were modified only in terms of the active‐source shape and panel dimensions. Specifically, *R*_2_ is changed to 0.1 m, *R*_3_ is changed to 0.15 m, and the shape of active source is altered from a circle with radius *R*_1_ to the corresponding desired shape.

### Experiments

The system was geometrically identical to the 3D numerical simulation, with details of the layer thicknesses provided in Table S1 (Supporting Information). The experimental structure was fabricated using metal 3D printing, with a processing time of 24 h and a precision of 0.05 mm. The circular plate has a radius of 12 cm, with a central transformation region of 8 cm in radius, intended for camouflaging the active source. The annular region between 8 and 12 cm radius serves as the background area to assess whether the ASM design affects the thermal field in the background region. The outermost ring was a water channel used to circulate constant‐temperature cooling water, establishing a low‐temperature boundary. A circular heating element with a diameter of 8 cm was positioned at the bottom to simulate the active source. In experiments, the current and voltage applied to the central heating element are ≈0.3 A and 170 V, respectively. Under these conditions, the heating power $q=10000\;W\cdot m^{-2}$ is consistently applied for each experimental set. Circulating water flows along the device's edges at a rate of 4 L min^−1^, with inlet and outlet temperatures of 20 and 20.4 °C, respectively. The experimental materials were uniformly made of the same aluminum alloy with a thermal conductivity of 176 W · m^−2^ · K^−1^. Their multi‐layered structure was detailed in the supplementary materials, outlining the design principles and layer configurations. To mitigate the impact of voids on infrared temperature measurements, PDMS is filled between each layer and the entire experimental board is coated with black ink to ensure uniform emissivity. Finally, to minimize thermal resistance between the active source and the thermal metamaterial, a thin layer of highly conductive soft material is inserted between them.

## Conflict of Interest

The authors declare no conflict of interest.

## Author Contributions

X.C., Z.T., and Y.N. contributed equally to this work. X.C. proposed the idea and theory; Y.L., X.C., and J.L. constructed the theory and performed the numerical simulations; Y.L., J.L., and X.C. designed the experiments; Y.L., X.C., J.L., Y.Z., Z.Z., Z.Z., D.J., and Z.T. performed the experiments; L.H., Z.C., X.C., and Y.L. wrote the manuscript. All the authors contributed to the manuscript editing; Z.C. and L.H. supervised the work.

## Supporting information



Supporting Information

## Data Availability

The data that support the findings of this study are available from the corresponding author upon reasonable request.
