# Supplementary material for: Inverse Design of Active‐Source Metamaterials for Thermal Camouflage with Arbitrary Active Sources
Source: Adv Sci (Weinh). 2025 Apr 15;12(26):2503024. doi: 10.1002/advs.202503024 (PMC12244515; doi:10.1002/advs.202503024)
Supplement: Supplementary file 1 — Supporting Information [file ADVS-12-2503024-s001.docx]

Supporting Information

Inverse Design of Active-Source Metamaterials for Thermal Camouflage with Arbitrary Active Sources

*Xianrong Cao* ^a,1^, *Zifeng Tong* ^a,1^, *Yongle Nian* ^a,1^, *Jiachang Li* ^a^, *Yinuo Zhou* ^a^, *Zihao Zhang* ^a^, *Yixin Liu* ^a^, *Lei Gong* ^b,^*, *Zhengdong Cheng* ^c,^*, *Liqun He* ^a,^*.

**S1.1: Inverse design framework of ASM**

In the main text, we present the solution procedure for the general solution of two-dimensional active-source thermal fields. In this section, we extend the approach to the case of three-dimensional active-source thermal fields. Leveraging the principles of the inverse heat conduction problem, the thermal conductivity can be derived from a known temperature field distribution. According to Fourier's law, the governing equation for heat conduction is expressed as:

$$\begin{aligned} \rho c\frac{\partial T}{\partial t}=\nabla\left( \kappa\nabla T \right)+q\#\left( S1 \right) \end{aligned}$$

Here, $\rho$, $c$, and $\kappa$ represent the material's density, specific heat capacity, and thermal conductivity, respectively. $T$, $t$, and $q$ denote temperature, time, and active source, respectively. In this context, we focus on the steady-state heat conduction problem where $\rho c{\partial T}/{\partial t}=0$. Therefore, the steady-state heat conduction equation with active sources, expanded in a three-dimensional Cartesian coordinate system, is given as:

$$\begin{aligned} \frac{\partial T}{\partial x}\frac{\partial\kappa}{\partial x}+\frac{\partial T}{\partial y}\frac{\partial\kappa}{\partial y}+\frac{\partial T}{\partial z}\frac{\partial\kappa}{\partial z}+\left( \frac{\partial^{2}T}{\partial x^{2}}+\frac{\partial^{2}T}{\partial y^{2}}+\frac{\partial^{2}T}{\partial z^{2}} \right)\kappa+q=0\#\left( S2 \right) \end{aligned}$$

It is important to highlight that, at this stage, the thermal conductivity $\kappa$ being solved for is assumed to be isotropic. For computational convenience, the expanded form of the equation can be restructured into a more general form as follows:

$$\begin{aligned} \sum_{j=1}^{n} b_{j}\frac{\partial u}{\partial x_{j}}+cu=f\#\left( S3 \right) \end{aligned}$$

In this equation, $u$ represents the thermal conductivity, and $f$ denotes the source term $q$. The coefficients $b_{j}=b_{j}(x_{1}, x_{2}, \ldots, x_{n})$ are associated with the first derivatives of $u$, where $j=1, 2, \cdots, n$ indicates the dimensionality. $c=c(x_{1}, x_{2}, \ldots, x_{n})$ represents the coefficients for the second derivatives of $T$. This constitutes an inhomogeneous equation. To derive the general solution for the thermal conductivity $u$, we first solve the homogeneous form of Equation (S3).

$$\begin{aligned} \sum_{j=1}^{n} b_{j}\frac{\partial u}{\partial x_{j}}+cu=0\#\left( S4 \right) \end{aligned}$$

However, current mathematical methods do not allow us to directly solve such an $n$-order partial differential equation. Consequently, we establish a system of characteristic equations.

$$\begin{aligned} \frac{dx_{1}}{b_{1}}=\frac{dx_{2}}{b_{2}}=\ldots=\frac{dx_{n}}{b_{n}}\#\left( S5 \right) \end{aligned}$$

When selecting one of $x_{1}, x_{2}, \cdots, x_{n}$ (such as $x_{n}$) as a parameter, the characteristic equations can be transformed into a system of $n-1$ first-order ordinary differential equations: ${dx_{j}}/{dx_{n}}={b_{j}}/{b_{n}}(j=1, 2,\cdots, n-1)$. According to the theory of ordinary differential equations, the solutions to this system of $n-1$ first-order ordinary differential equations can be expressed as $\varphi\left( x_{1}, x_{2}, \ldots, x_{n} \right)=h_{j}\left( j=1,2,\ldots,n-1 \right)$, which represent the $n-1$ independent first integrals of equation (S5).

Next, using these $n-1$ independent first integrals as variables, we perform a variable substitution.

$$\begin{aligned} \left\{ \begin{aligned} \xi_{j}=&\varphi_{j}\left( x_{1}, x_{2}, \ldots, x_{n} \right), j=1,2,\ldots,n-1 \\ \xi_{n}=&\varphi_{n}\left( x_{1}, x_{2}, \ldots, x_{n} \right) \end{aligned} \right.\#\left( S6 \right) \end{aligned}$$

Here, $\varphi_{n}\left( x_{1}, x_{2}, \ldots, x_{n} \right)$ can be chosen arbitrarily, provided it is linearly independent of $\varphi_{j}$.

$$\begin{aligned} J\left( \varphi_{1}, \varphi_{2}, \ldots, \varphi_{n} \right)=\frac{\partial\left( \varphi_{1}, \varphi_{2}, \ldots, \varphi_{n} \right)}{\partial\left( x_{1}, x_{2}, \ldots, x_{n} \right)}=\left| \begin{matrix} \frac{\partial\varphi_{1}}{\partial x_{1}} & \frac{\partial\varphi_{1}}{\partial x_{2}} & \cdots& \frac{\partial\varphi_{1}}{\partial x_{n}} \\ \frac{\partial\varphi_{2}}{\partial x_{1}} & \frac{\partial\varphi_{2}}{\partial x_{2}} & \cdots& \frac{\partial\varphi_{2}}{\partial x_{n}} \\ \vdots& \vdots& \ddots& \vdots\\ \frac{\partial\varphi_{n}}{\partial x_{1}} & \frac{\partial\varphi_{n}}{\partial x_{2}} & \cdots& \frac{\partial\varphi_{n}}{\partial x_{n}} \end{matrix} \right|\neq0\#\left( S7 \right) \end{aligned}$$

Equation (S7) ensures that the new variables $\varphi_{1}, \varphi_{2}, \ldots, \varphi_{n}$ satisfy mutually independent relationships. Substituting them into equation (S3), we apply the chain rule to obtain:

$$\begin{aligned} \sum_{j=1}^{n} b_{j}\frac{\partial u}{\partial x_{j}}=\sum_{j=1}^{n} b_{j}\left( \sum_{i=1}^{n} \frac{\partial\varphi_{i}}{\partial x_{j}}\frac{\partial u}{\partial\xi_{i}} \right)=\sum_{i=1}^{n} \left( \sum_{j=1}^{n} b_{j}\frac{\partial\varphi_{i}}{\partial x_{j}} \right)\frac{\partial u}{\partial\xi_{i}}\#\left( S8 \right) \end{aligned}$$

According to the theorem of partial differential equations, when $i=1, 2,\cdots, n-1$, the equation

$$\begin{aligned} \sum_{j=1}^{n} b_{j}\frac{\partial\varphi_{i}}{\partial x_{j}}=0\#\left( S9 \right) \end{aligned}$$

Therefore, equation (S3) can be written as

$$\begin{aligned} \left( \sum_{j=1}^{n} b_{j}\frac{\partial\varphi_{n}}{\partial x_{j}} \right)\frac{\partial u}{\partial\xi_{n}}+cu=f, u=u\left( \xi_{1}, \xi_{2}, \ldots, \xi_{n} \right)\#\left( S10 \right) \end{aligned}$$

The general solution of equation (S10) can be obtained by integrating with respect to $\xi_{n}$.

$$\begin{aligned} u=\exp\left( -\int\frac{c}{\sum_{j=1}^{n} b_{j}\frac{\partial\varphi_{n}}{\partial\xi_{n}}}d\xi_{n} \right)\cdot\left[ \int\exp\left( \int\frac{c}{\sum_{j=1}^{n} b_{j}\frac{\partial\varphi_{n}}{\partial\xi_{n}}}d\xi_{n} \right)\frac{f}{\sum_{j=1}^{n} b_{j}\frac{\partial\varphi_{n}}{\partial\xi_{n}}}+g\left( \xi_{1}, \xi_{2}, \ldots, \xi_{n} \right) \right]\#\left( S11 \right) \end{aligned}$$

Here, $g=g (\xi_{1}, \xi_{2}, \ldots, \xi_{n-1})$ is a function of $\xi_{1}, \xi_{2}, \ldots, \xi_{n-1}$. For simplicity, we define:

$$\begin{aligned} \mu=\frac{c}{\sum_{j=1}^{n} b_{j}\frac{\partial\varphi_{n}}{\partial\xi_{n}}}, \nu=\frac{f}{\sum_{j=1}^{n} b_{j}\frac{\partial\varphi_{n}}{\partial\xi_{n}}}\#\left( S12 \right) \end{aligned}$$

The general solution can be simplified to

$$\begin{aligned} u=\frac{\int\nu e^{\int\mu d\xi_{n}}d\xi_{n}+g}{e^{\int\mu d\xi_{n}}}\#\left( S13 \right) \end{aligned}$$

The general formula obtained at this step provides a powerful mathematical tool for precisely controlling active-source thermal fields. Next, we will provide a detailed derivation of the general solution of the steady-state heat conduction equation in two-dimensional polar coordinates, which is expressed as follows:

$$\begin{aligned} \frac{1}{r}\frac{\partial\left( \kappa r\frac{\partial T}{\partial r} \right)}{\partial r}+\frac{1}{r^{2}}\frac{\partial\left( \kappa\frac{\partial T}{\partial\theta} \right)}{\partial\theta}+q=0\#\left( S14 \right) \end{aligned}$$

Here, $q$ represents the source term, while $r$ and $\theta$ denote the radial and angular coordinates, respectively. As previously discussed, by introducing the substitution variables $\varepsilon=\varphi(r, \theta)$ and $\omega=\phi(r, \theta)$, equation (S14) can be rewritten and simplified using the chain rule.

$$\begin{aligned} \left( rf_{1}\frac{\partial\varphi}{\partial r}+\frac{1}{r}f_{2}\frac{\partial\varphi}{\partial\theta} \right)\frac{\partial\kappa}{\partial\varepsilon}+\left( rf_{1}\frac{\partial\phi}{\partial r}+\frac{1}{r}f_{2}\frac{\partial\phi}{\partial\theta} \right)\frac{\partial\kappa}{\partial\omega}+\left( f_{1}+rf_{3}+\frac{f_{4}}{r} \right)\kappa+rq=0\#\left( S15 \right) \end{aligned}$$

where $f_{1}={\partial T}/{\partial r}$, $f_{2}={\partial T}/{\partial\theta}$, $f_{3}={\partial^{2}T}/{\partial r^{2}}$, and $f_{4}={\partial^{2}T}/{\partial\theta^{2}}$. To transform the second-order nonlinear partial differential equation into a first-order nonlinear partial differential equation, we define $\omega=\phi(r, \theta)$ such that it satisfies the following condition:

$$\begin{aligned} rf_{1}\frac{\partial\phi}{\partial r}+\frac{1}{r}f_{2}\frac{\partial\phi}{\partial\theta}=0\#\left( S16 \right) \end{aligned}$$

Thus, equation (S15) transforms into a first-order partial differential equation in terms of the variable $\varepsilon$. We can solve equation (S16) to obtain $\omega=f(r, \theta)$. Subsequently, we seek an $\varepsilon$ function that satisfies the following condition:

$$\begin{aligned} J\left( \varepsilon,\omega\right)=\frac{\partial\left( \varphi,\phi\right)}{\partial\left( r,\theta\right)}\neq0\#\left( S17 \right) \end{aligned}$$

Substituting $\varepsilon$ and $\omega$ into equation (S15) yields:

$$\begin{aligned} \frac{\partial\kappa}{\partial\varepsilon}+\frac{f_{1}+rf_{3}+\frac{f_{4}}{r}}{rf_{1}\frac{\partial\phi}{\partial r}+\frac{1}{r}f_{2}\frac{\partial\phi}{\partial\theta}}\kappa+\frac{rq}{rf_{1}\frac{\partial\varphi}{\partial r}+\frac{1}{r}f_{2}\frac{\partial\varphi}{\partial\theta}}=0\#\left( S18 \right) \end{aligned}$$

Similarly, let $\mu$ and $\nu$ represent the linear coefficient and the non-homogeneous term in equation (S18), respectively. Thus, the solution to equation (S18) can be expressed as:

$$\begin{aligned} \kappa=\frac{\int\nu e^{\int\mu d\omega}d\omega+f\left( \varepsilon\right)}{e^{\int\mu d\omega}}\#\left( S19 \right) \end{aligned}$$

where $f\left( \varepsilon\right)$ is a function of $\varepsilon$.

**S1.2: The active source distribution in coordinate transformations.**

While we have derived the theoretical formula for solving thermal conductivity in the presence of active sources, a crucial issue remains unaddressed. Specifically, during coordinate transformations, the source term in the equation will adjust accordingly (as illustrated in Figure S1). This means that we cannot directly use the active source from the target thermal fields in the predefined thermal fields. Thus, it becomes necessary to establish the relationship between the active-source distribution in the target thermal fields and its corresponding distribution in the predefined thermal fields.

Here we present the coordinate transformation relationship of the active sources under two-dimensional conditions as:

$$\begin{aligned} \iint_{D} Q\left( r,\theta\right)dS=\iint_{D^{'}} Q^{'}\left( r^{'},\theta^{'} \right)dS^{'}\#\left( S20 \right) \end{aligned}$$

In this context, regions *D* and *D*′ respectively represent the active-source regions before and after the coordinate transformation. $Q\left( r,\theta\right)$, $dS$ denote the heat power and differential area element in the original coordinate system, while $Q^{'}\left( r^{'},\theta^{'} \right)$, $dS^{'}$ represent the heat power and differential area element in the transformed coordinate system. Notably, by applying differential form transformations, we can derive a more intuitive relationship between active sources:

$$\begin{aligned} Qdrd\theta=Q^{'}dr^{'}d\theta^{'}\#\left( S20.1 \right) \end{aligned}$$

$$\begin{aligned} Q^{'}=\frac{Qdrd\theta}{dr^{'}d\theta^{'}}=\frac{Q}{\det\left( J \right)}\#\left( S20.2 \right) \end{aligned}$$

Given that the actual active source used, including its power and location, is known in the target thermal fields, we can derive the distribution of active sources in the predefined simple thermal fields using the above equation. Finally, formula (S19) allows us to obtain the distribution of thermal conductivity in the predefined thermal fields.

**S2:** **Derivation of theoretical models and transformation equations for active-source camouflage**

Here, we will provide a detailed derivation of the theoretical formulas of the model as presented in the main text. The transformation process of the designed model is illustrated in Figure S2, where the circular active-source region is radially compressed to obtain triangular, square, and cloverleaf shapes.

The predefined temperature field can be uniformly represented as

$$\begin{aligned} T=\left\{ \begin{aligned} &T_{1}+\frac{T_{2}-T_{1}}{R_{2}-R_{1}}\left( r-R_{1} \right), &R_{1}\leq r<R_{2}& \\ &T_{1},&r<R_{1}& \end{aligned} \right.\#\left( S21 \right) \end{aligned}$$

where $R_{1}$ and $R_{2}$ represent the radii of the inner and outer circles, respectively. $r$ denotes the distance from a point inside the circle to the center. $T_{2}$ is the temperature within the camouflaged active-source region, while $T_{1}$ is the temperature on the outer boundary (as shown in Figure S2). Similarly, the target temperature field can also be expressed as：

$$\begin{aligned} T^{'}=\left\{ \begin{aligned} &T_{1}+\frac{T_{2}-T_{1}}{R_{2}-r_{1}}\left( r^{'}-r_{1} \right), r_{1}\leq&r^{'}<R_{2}& \\ &T_{1}, &r^{'}<r_{1} \end{aligned} \right.\#\left( S22 \right) \end{aligned}$$

where $r_{1}=r_{1}(\theta)$ represents the boundary of the camouflaged active-source shape. By determining the coordinate transformation relationship between the predefined temperature field and the target temperature field, the annular region $R_{1}<r<R_{2}$ is transformed into the region between $r_{1}\left( \theta\right)<r<R_{2}$, as shown in Figure S2. The corresponding transformation formula is:

$$\begin{aligned} \left\{ \begin{aligned} &r^{'}=\frac{R_{2}-r_{1}}{R_{2}-R_{1}}\left( r-R_{1} \right)+r_{1} \\ &\theta^{'}=\theta\end{aligned} \right.\#\left( S23 \right) \end{aligned}$$

Due to $\theta^{'}=\theta$, which implies that the spatial transformation occurs only along the radial direction, the magnitude and direction of heat flux remain unchanged before and after the coordinate transformation. Next, we will conduct a detailed analysis for each model.

**S2.1: Thermal conductivity tensor for triangular camouflage of a circular active source.**

As shown in Figure S2**a**, the boundary line of the triangle is

$$\begin{aligned} r_{1}\left( \theta\right)=\left\{ \begin{aligned} &\frac{R_{1}}{2\cos\left( \theta\right)}, -\frac{\pi}{3}<\theta\leq\frac{\pi}{3} \\ &\frac{R_{1}}{2\cos\left( \theta-\frac{2}{3}\pi\right)}, \frac{\pi}{3}<\theta\leq\pi\\ &\frac{R_{1}}{2\cos\left( \theta+\frac{2}{3}\pi\right)}, -\pi<\theta\leq-\frac{\pi}{3}& \end{aligned} \right.\#\left( S24 \right) \end{aligned}$$

The coordinate transformation formula corresponding to region A is

$$\begin{aligned} \left\{ \begin{aligned} &r^{'}=\frac{{2R}_{2}\cos\left( \theta\right)-R_{1}}{2\left( R_{2}-R_{1} \right)\cos\left( \theta\right)}\left( r-R_{1} \right)+\frac{R_{1}}{2\cos\left( \theta\right)} \\ &\theta^{'}=\theta\end{aligned} \right.\#\left( S25 \right) \end{aligned}$$

The corresponding coordinate transformation matrix is

$$\begin{aligned} \boldsymbol{J}_{\boldsymbol{1}}\boldsymbol{=}\left( \begin{matrix} \frac{{2R}_{2}\cos\left( \theta\right)-R_{1}}{2\left( R_{2}-R_{1} \right)\cos\left( \theta\right)} & \frac{1}{r}\frac{R_{2}-r}{R_{2}-R_{1}}\frac{R_{1}\sin\theta}{\cos^{2} \theta} \\ 0 & \frac{{2R}_{2}\cos\left( \theta\right)-R_{1}}{2\left( R_{2}-R_{1} \right)\cos\left( \theta\right)}\frac{r-R_{1}}{r}+\frac{R_{1}}{2r \cos\left( \theta\right)} \end{matrix} \right)\boldsymbol{\#}\left( S26 \right) \end{aligned}$$

Similarly, the coordinate transformation formula corresponding to region B is

$$\begin{aligned} \left\{ \begin{aligned} &r^{'}=\frac{r}{2\cos\left( \theta\right)} \\ &\theta^{'}=\theta\end{aligned} \right.\#\left( S27 \right) \end{aligned}$$

The corresponding coordinate transformation matrix is

$$\begin{aligned} \boldsymbol{J}_{\boldsymbol{2}}\boldsymbol{=}\left( \begin{matrix} \frac{1}{2\cos\left( \theta\right)} & 0 \\ 0 & \frac{{2R}_{2}\cos\left( \theta\right)-R_{1}}{2\left( R_{2}-R_{1} \right)\cos\left( \theta\right)}\frac{r-R_{1}}{r}+\frac{R_{1}}{2r \cos\left( \theta\right)} \end{matrix} \right)\boldsymbol{\#}\left( S28 \right) \end{aligned}$$

According to equation (S20), in the target thermal fields, the active source is a circular active source with radius $R_{1}$ and constant power $Q_{0}$. Therefore, in the predefined thermal fields, the power of active source satisfies

$$\begin{aligned} Q\left( r,\theta\right)=\left\{ \begin{aligned} &Q_{0}\frac{{2R}_{2}\cos\left( \theta\right)-R_{1}}{2{{(R}_{2}-R_{1})}^{2}r\cos\left( \theta\right)}\left( \frac{{2R}_{2}\cos\left( \theta\right)-R_{1}}{2\left( R_{2}-R_{1} \right)\cos\left( \theta\right)}\left( r-R_{1} \right)+\frac{R_{1}}{2\cos\left( \theta\right)} \right),R_{1}<&r<r_{2} \\ &\frac{Q_{0}}{4\cos^{2} \left( \theta\right)},&r<R_{1} \end{aligned} \right.\#\left( S29 \right) \end{aligned}$$

where $r_{2}=(R_{1}-r_{1})/(R_{2}-r_{1})\left( R_{2}-R_{1} \right)+R_{1}$ represents the boundary line of the shape obtained by the inverse transformation of a circle with radius $R_{1}$, as shown in Figure S3**a**. Region E satisfies $R_{1}<r<r_{2}$, containing the active source, while region F satisfies $r_{2}<r<R_{2}$ and does not contain the active source.

The thermal conductivity in region E is

$$\begin{aligned} \begin{aligned} \kappa\left( r,\theta\right)=&\frac{Q_{0}R_{1}^{2}}{8\frac{T_{2}-T_{1}}{R_{2}-R_{1}}r\cos^{2} \left( \theta\right)} \\ &+\frac{Q_{0}}{\frac{T_{2}-T_{1}}{R_{2}-R_{1}}r}\left( \frac{1}{8}\left( \frac{{2R}_{2}\cos\left( \theta\right)-R_{1}}{\left( R_{2}-R_{1} \right)\cos\left( \theta\right)} \right)^{2}\left( r^{2}-R_{1}^{2} \right)+\frac{R_{1}R_{2}\left( 1-2\cos\left( \theta\right) \right)}{2\left( R_{2}-R_{1} \right)\cos\left( \theta\right)}\left( r-R_{1} \right) \right)\# \end{aligned}\#\left( S30 \right) \end{aligned}$$

The thermal conductivity in region F is

$$\begin{aligned} \kappa\left( r,\theta\right)=&\frac{Q_{0}R_{1}^{2}}{8\frac{T_{2}-T_{1}}{R_{2}-R_{1}}r\cos^{2} \left( \theta\right)} \\ &+\frac{Q_{0}}{\frac{T_{2}-T_{1}}{R_{2}-R_{1}}r}\left( \begin{aligned} \frac{1}{8}\left( \frac{{2R}_{2}\cos\left( \theta\right)-R_{1}}{\left( R_{2}-R_{1} \right)\cos\left( \theta\right)} \right)^{2}\left( \left( \frac{R_{1}-\frac{R_{1}}{2\cos\left( \theta\right)}}{R_{2}-\frac{R_{1}}{2\cos\left( \theta\right)}}\left( R_{2}-R_{1} \right)+R_{1} \right)^{2}-R_{1}^{2} \right) \\ -{R_{1}^{2}R}_{2}\frac{\left( 1-2\cos\left( \theta\right) \right)^{2}}{{4R}_{2}\cos^{2} \left( \theta\right)-2R_{1}\cos\left( \theta\right)} \end{aligned} \right)\#\left( S31 \right) \end{aligned}$$

Based on the coordinate transformation theory^[1,2]^, the thermal conductivity distribution after transformation can be calculated as

$$\begin{aligned} \kappa_{r^{'}r^{'}}=\kappa\left( r,\theta\right)\left[ \begin{aligned} \frac{{2R}_{2}\cos\left( \theta\right)-R_{1}}{2\left( R_{2}-R_{1} \right)\cos\left( \theta\right)}\left( \frac{{2R}_{2}\cos\left( \theta\right)-R_{1}}{2\left( R_{2}-R_{1} \right)\cos\left( \theta\right)}\frac{r-R_{1}}{r}+\frac{R_{1}}{2r \cos\left( \theta\right)} \right) \\ +\frac{R_{1}^{2}\left( R_{2}-r \right)^{2}\sin^{2} \left( \theta\right)}{2\left( R_{2}-R_{1} \right)\left( {2R}_{2}\cos\left( \theta\right)-R_{1} \right)\left( \frac{{2R}_{2}\cos\left( \theta\right)-R_{1}}{2\left( R_{2}-R_{1} \right)}\left( r-R_{1} \right)+\frac{R_{1}}{2} \right)\cos^{4} \left( \theta\right)}\frac{1}{r} \end{aligned} \right]\#\left( S32a \right) \end{aligned}$$

$$\begin{aligned} \kappa_{r^{'}\theta^{'}}=\kappa_{\theta^{'}r^{'}}=\kappa\left( r,\theta\right)\frac{R_{1}\left( R_{2}-r \right)\tan\left( \theta\right)}{R_{2}\cos\left( \theta\right)-\frac{R_{1}}{2}}\frac{1}{r}\#\left( S32b \right) \end{aligned}$$

$$\begin{aligned} \kappa_{\theta^{'}\theta^{'}}=\kappa\left( r,\theta\right)\frac{R_{2}-r}{R_{2}\cos\left( \theta\right)-\frac{R_{1}}{2}}\left( \frac{{2R}_{2}\cos\left( \theta\right)-R_{1}}{2\left( R_{2}-R_{1} \right)}\frac{r-R_{1}}{r}+\frac{R_{1}}{2r} \right)\#\left( S32c \right) \end{aligned}$$

When $r<r_{1}$, the thermal conductivity is set to $\kappa=\kappa_{0}$, where $\kappa_{0}$ is significantly higher than the thermal conductivity in other regions. This results in a nearly isothermal condition within this area, achieving the desired camouflage. Given a background temperature of 293 K, a predefined high temperature in the active-source region of $T_{2}=$400 K, a active-source power of 10000$W\cdot m^{-2}$, and radii $R_{1}=0.05 m$ and $R_{2}=0.10 m$, the final thermal conductivity distribution is shown in Figure S4**a**.

**S2.2: Thermal conductivity tensor for square camouflage of a circular active source.**

Next, we will camouflage the circular active source as a square. The boundary line of the square is

$$\begin{aligned} r_{1}\left( \theta\right)=\frac{R_{1}}{\sqrt{2}max(|cos(\theta)|,|sin(\theta)|)}\#\left( S33 \right) \end{aligned}$$

Considering the high symmetry of the square, we demonstrate the design process for the region $\pi/4<\theta<{3\pi}/4$. The thermal conductivity tensor for other regions can be obtained similarly. As shown in Figure S2**b**, the coordinate transformation formula for region A is

$$\begin{aligned} \left\{ \begin{aligned} &r^{'}=\frac{\sqrt{2}R_{2}\sin\left( \theta\right)-R_{1}}{\sqrt{2}\left( R_{2}-R_{1} \right)\sin\left( \theta\right)}\left( r-R_{1} \right)+\frac{R_{1}}{\sqrt{2}\sin\left( \theta\right)} \\ &\theta^{'}=\theta\end{aligned} \right.\#\left( S34 \right) \end{aligned}$$

The corresponding transformation matrix is

$$\begin{aligned} \boldsymbol{J}_{1}\boldsymbol{=}\left( \begin{matrix} \frac{\sqrt{2}R_{2}\sin\left( \theta\right)-R_{1}}{\sqrt{2}\left( R_{2}-R_{1} \right)\sin\left( \theta\right)} & -\frac{1}{r}\frac{R_{2}-r}{R_{2}-R_{1}}\frac{R_{1}\cos\left( \theta\right)}{\sqrt{2}\sin^{2} \left( \theta\right)} \\ 0 & \frac{\sqrt{2}R_{2}\sin\left( \theta\right)-R_{1}}{\sqrt{2}\left( R_{2}-R_{1} \right)\sin\left( \theta\right)}\frac{r-R_{1}}{r}+\frac{R_{1}}{\sqrt{2}r\sin\left( \theta\right)} \end{matrix} \right)\boldsymbol{\#}\left( S35 \right) \end{aligned}$$

The coordinate transformation formula for region B is

$$\begin{aligned} \left\{ \begin{aligned} &r^{'}=\frac{r}{\sqrt{2}\sin\left( \theta\right)} \\ &\theta^{'}=\theta\end{aligned} \right.\#\left( S36 \right) \end{aligned}$$

The corresponding transformation matrix is

$$\begin{aligned} \boldsymbol{J}_{2}\boldsymbol{=}\left( \begin{matrix} \frac{1}{\sqrt{2}\sin\left( \theta\right)} & 0 \\ 0 & \frac{\sqrt{2}R_{2}\sin\left( \theta\right)-R_{1}}{\sqrt{2}\left( R_{2}-R_{1} \right)\sin\left( \theta\right)}\frac{r-R_{1}}{r}+\frac{R_{1}}{\sqrt{2}r\sin\left( \theta\right)} \end{matrix} \right)\boldsymbol{\#}\left( S37 \right) \end{aligned}$$

According to equation (S20), if the circular active source after coordinate transformation has a radius $R_{1}$ and a constant power $Q_{0}$, then the active-source power distribution before the transformation satisfies

$$\begin{aligned} \begin{aligned} Q\left( r,\theta\right)=\left\{ \begin{aligned} &Q_{0}\frac{{2R}_{2}\cos\left( \theta\right)-R_{1}}{2{{(R}_{2}-R_{1})}^{2}r\cos\left( \theta\right)}\left( \frac{{2R}_{2}\cos\left( \theta\right)-R_{1}}{2\left( R_{2}-R_{1} \right)\cos\left( \theta\right)}\left( r-R_{1} \right)+\frac{R_{1}}{2\cos\left( \theta\right)} \right),R_{1}\leq&r<r_{2} \\ &\frac{Q_{0}}{4\cos^{2} (\theta)}, &r<R_{1} \end{aligned} \right.\#\left( S38 \right) \end{aligned} \end{aligned}$$

where $r_{2}=(R_{1}-r_{1})/(R_{2}-r_{1})\left( R_{2}-R_{1} \right)+R_{1}$ represents the boundary line of the shape obtained by the inverse transformation of a circle with radius $R_{1}$, as shown in Figure S3**b**. In region E, $R_{1}<r<r_{2}$, containing the active source, while region F, $r_{2}<r<R_{2}$, does not contain the active source.

The thermal conductivity in region E is

$$\begin{aligned} \begin{aligned} \kappa\left( r,\theta\right)=&\frac{Q_{0}R_{1}^{2}}{4\frac{T_{2}-T_{1}}{R_{2}-R_{1}}r\sin^{2} \left( \theta\right)} \\ &+\frac{Q_{0}}{\frac{T_{2}-T_{1}}{R_{2}-R_{1}}r}\left( \frac{1}{4}\left( \frac{{\sqrt{2}R}_{2}\sin\left( \theta\right)-R_{1}}{\left( R_{2}-R_{1} \right)\sin\left( \theta\right)} \right)^{2}\left( r^{2}-R_{1}^{2} \right)+\frac{R_{1}R_{2}\left( 1-\sqrt{2}\sin\left( \theta\right) \right)}{\sqrt{2}\left( R_{2}-R_{1} \right)\sin\left( \theta\right)}\left( r-R_{1} \right) \right)\# \end{aligned}\#\left( S39 \right) \end{aligned}$$

The thermal conductivity distribution in region F is

$$\begin{aligned} \kappa\left( r,\theta\right)&=\frac{Q_{0}R_{1}^{2}}{4\frac{T_{2}-T_{1}}{R_{2}-R_{1}}r\sin^{2} \left( \theta\right)} \\ &+\frac{Q_{0}}{\frac{T_{2}-T_{1}}{R_{2}-R_{1}}r}\left( \begin{aligned} \frac{1}{4}\left( \frac{{\sqrt{2}R}_{2}\sin\left( \theta\right)-R_{1}}{\left( R_{2}-R_{1} \right)\sin\left( \theta\right)} \right)^{2}\left( \left( \frac{R_{1}-\frac{R_{1}}{\sqrt{2}\sin\left( \theta\right)}}{R_{2}-\frac{R_{1}}{\sqrt{2}\sin\left( \theta\right)}}\left( R_{2}-R_{1} \right)+R_{1} \right)^{2}-R_{1}^{2} \right) \\ -{R_{1}^{2}R}_{2}\frac{\left( 1-\sqrt{2}\sin\left( \theta\right) \right)^{2}}{2R_{2}\sin^{2} \left( \theta\right)-\sqrt{2}R_{1}\sin\left( \theta\right)} \end{aligned} \right)\#\left( S40 \right) \end{aligned}$$

Based on the coordinate transformation theory^[3,4]^, the thermal conductivity tensor is

$$\begin{aligned} &\kappa_{r^{'}r^{'}} \\ &=\kappa\left( r,\theta\right)\left[ \begin{aligned} \frac{{\sqrt{2}R}_{2}\sin\left( \theta\right)-R_{1}}{\sqrt{2}\left( R_{2}-R_{1} \right)\sin\left( \theta\right)}\left( \frac{{\sqrt{2}R}_{2}\sin\left( \theta\right)-R_{1}}{\sqrt{2}\left( R_{2}-R_{1} \right)\sin\left( \theta\right)}\frac{r-R_{1}}{r}+\frac{R_{1}}{\sqrt{2}r \sin\left( \theta\right)} \right) \\ +\frac{{R_{1}^{2}\left( R_{2}-r \right)}^{2}\cos^{2} \left( \theta\right)}{\sqrt{2}\left( R_{2}-R_{1} \right)\left( \sqrt{2}R_{2}\sin\left( \theta\right)-R_{1} \right)\left( \frac{\sqrt{2}R_{2}\sin\left( \theta\right)-R_{1}}{\sqrt{2}\left( R_{2}-R_{1} \right)}\left( r-R_{1} \right)+\frac{R_{1}}{\sqrt{2}} \right)\sin^{4} \left( \theta\right)}\frac{1}{r} \end{aligned} \right]\#\left( S41a \right) \end{aligned}$$

$$\begin{aligned} \kappa_{r^{'}\theta^{'}}=\kappa_{\theta^{'}r^{'}}=\kappa\left( r,\theta\right)\frac{R_{1}\left( R_{2}-r \right)\cot\left( \theta\right)}{R_{2}\sin\left( \theta\right)-\frac{R_{1}}{\sqrt{2}}}\frac{1}{r}\#\left( S41b \right) \end{aligned}$$

$$\begin{aligned} \kappa_{\theta^{'}\theta^{'}}=\kappa\left( r,\theta\right)\frac{R_{2}-r}{R_{2}\sin\left( \theta\right)-\frac{R_{1}}{\sqrt{2}}}\left( \frac{{\sqrt{2}R}_{2}\sin\left( \theta\right)-R_{1}}{\sqrt{2}\left( R_{2}-R_{1} \right)}\frac{r-R_{1}}{r}+\frac{R_{1}}{\sqrt{2}r} \right)\#\left( S41c \right) \end{aligned}$$

Similarly, when $r<r_{1}$, the thermal conductivity is set to $\kappa$ = $\kappa_{0}$. The resulting thermal conductivity distribution is shown in Figure S4**b**.

**S2.3: Thermal conductivity tensor for cloverleaf camouflage of the circular active source.**

The boundary line of the cloverleaf shape is

$$\begin{aligned} r_{1}\left( \theta\right)=\frac{R_{1}}{5}\left( \sin\left( 3\theta\right)+2 \right)\#\left( S42 \right) \end{aligned}$$

Similarly, as shown in Figure S2**c**, the coordinate transformation formula for region A is

$$\begin{aligned} \left\{ \begin{aligned} &r^{'}=\frac{R_{2}-\frac{R_{1}}{5}\left( \sin\left( 3\theta\right)+2 \right)}{R_{2}-R_{1}}\left( r-R_{1} \right)+\frac{R_{1}}{5}\left( \sin\left( 3\theta\right)+2 \right) \\ &\theta^{'}=\theta\end{aligned} \right.\#\left( S43 \right) \end{aligned}$$

The corresponding transformation matrix is

$$\begin{aligned} \boldsymbol{J}_{1}\boldsymbol{=}\left( \begin{matrix} \frac{R_{2}-\frac{R_{1}}{5}\left( \sin\left( 3\theta\right)+2 \right)}{R_{2}-R_{1}} & \frac{1}{r}\frac{R_{2}-r}{R_{2}-R_{1}}\frac{3R_{1}}{5}\cos\left( 3\theta\right) \\ 0 & \frac{R_{2}-\frac{R_{1}}{5}\left( \sin\left( 3\theta\right)+2 \right)}{R_{2}-R_{1}}\frac{\left( r-R_{1} \right)}{r}+\frac{R_{1}}{5}\frac{\left( \sin\left( 3\theta\right)+2 \right)}{r} \end{matrix} \right)\boldsymbol{\#} \end{aligned}(S44)$$

The coordinate transformation formula for region B is

$$\begin{aligned} \left\{ \begin{aligned} &r^{'}=\frac{\sin\left( 3\theta\right)+2}{5}r \\ &\theta^{'}=\theta\end{aligned} \right.\#\left( S45 \right) \end{aligned}$$

The corresponding transformation matrix is

$$\begin{aligned} \boldsymbol{J}_{2}\boldsymbol{=}\left( \begin{matrix} \frac{\left( \sin\left( 3\theta\right)+2 \right)}{5} & 0 \\ 0 & \frac{R_{2}-\frac{R_{1}}{5}\left( \sin\left( 3\theta\right)+2 \right)}{R_{2}-R_{1}}\frac{\left( r-R_{1} \right)}{r}+\frac{R_{1}}{5}\frac{\left( \sin\left( 3\theta\right)+2 \right)}{r} \end{matrix} \right)\boldsymbol{\#} \end{aligned}(S46)$$

Similarly, from equation (S20), it can be inferred that the active-source power in the predefined thermal fields satisfies

$$\begin{aligned} &Q\left( r,\theta\right) \\ &=\left\{ \begin{aligned} &Q_{0}\frac{R_{2}-\frac{R_{1}}{5}\left( \sin\left( 3\theta\right)+2 \right)}{{(R}_{2}-R_{1})r}\left( \begin{aligned} \frac{R_{2}-\frac{R_{1}}{5}\left( \sin\left( 3\theta\right)+2 \right)}{R_{2}-R_{1}}\left( r-R_{1} \right) \\ +\frac{R_{1}}{5}\left( \sin\left( 3\theta\right)+2 \right) \end{aligned} \right),R_{1}<&r<r_{2} \\ &Q_{0}\frac{R_{1}^{2}\left( \sin\left( 3\theta\right)+2 \right)^{2}}{25R_{2}}, &r<R_{1} \end{aligned} \right.\#\left( S47 \right) \end{aligned}$$

where $r_{2}=(R_{1}-r_{1})/(R_{2}-r_{1})\left( R_{2}-R_{1} \right)+R_{1}$. The boundary line $r_{2}$ defines the cloverleaf shape obtained through the inverse transformation of a circle with radius $R_{1}$, as shown in Figure S3**c**. Region E ($R_{1}<r<r_{2}$) contains the active source, whereas region F ($r_{2}<r<R_{2}$) does not.

The thermal conductivity in region E is

$$\begin{aligned} &\kappa\left( r,\theta\right) \\ &=\frac{Q_{0}\left( R_{1}\left( \sin\left( 3\theta\right)+2 \right) \right)^{2}}{50\frac{T_{2}-T_{1}}{R_{2}-R_{1}}r} \\ &+\frac{Q_{0}}{\frac{T_{2}-T_{1}}{R_{2}-R_{1}}r}\left( \frac{1}{2}\left( \frac{R_{2}-\frac{R_{1}}{5}\left( \sin\left( 3\theta\right)+2 \right)}{R_{2}-R_{1}} \right)^{2}\left( r^{2}-R_{1}^{2} \right)+R_{2}\frac{\frac{R_{1}}{5}\left( \sin\left( 3\theta\right)+2 \right)-R_{1}}{R_{2}-R_{1}}\left( r-R_{1} \right) \right)\#\left( S48 \right) \end{aligned}$$

The thermal conductivity distribution in region F is

$$\begin{aligned} &\kappa\left( r,\theta\right) \\ &=\frac{Q_{0}\left( R_{1}\left( \sin\left( 3\theta\right)+2 \right) \right)^{2}}{50\frac{T_{2}-T_{1}}{R_{2}-R_{1}}r} \\ &+\frac{Q_{0}}{\frac{T_{2}-T_{1}}{R_{2}-R_{1}}r}\left( \begin{aligned} \frac{1}{2}\left( \frac{R_{2}-\frac{R_{1}}{5}\left( \sin\left( 3\theta\right)+2 \right)}{R_{2}-R_{1}} \right)^{2}\left( \left( \frac{R_{1}-\frac{R_{1}}{5}\left( \sin\left( 3\theta\right)+2 \right)}{R_{2}-\frac{R_{1}}{5}\left( \sin\left( 3\theta\right)+2 \right)}\left( R_{2}-R_{1} \right)+R_{1} \right)^{2}-R_{1}^{2} \right) \\ +R_{2}\frac{\left( \frac{R_{1}}{5}\left( \sin\left( 3\theta\right)+2 \right)-R_{1} \right)\left( R_{1}-\frac{R_{1}}{5}\left( \sin\left( 3\theta\right)+2 \right) \right)}{R_{2}-\frac{R_{1}}{5}\left( \sin\left( 3\theta\right)+2 \right)} \end{aligned} \right)\#\left( S49 \right) \end{aligned}$$

Based on the coordinate transformation theory^[5,6]^, the thermal conductivity tensor is

$$\begin{aligned} \kappa_{r^{'}r^{'}}=\kappa\left( r,\theta\right)\left[ \alpha_{1}+\alpha_{2}\alpha_{3} \right]\#\left( S50a \right) \end{aligned}$$

$$\begin{aligned} \kappa_{r^{'}\theta^{'}}=\kappa_{\theta^{'}r^{'}}=\kappa\left( r,\theta\right)\frac{3R_{1}{(R}_{2}-r)cos(3\theta)}{5\left( R_{2}-\frac{R_{1}}{5}\left( \sin\left( 3\theta\right)+2 \right) \right)}\frac{1}{r}\#\left( S50b \right) \end{aligned}$$

$$\begin{aligned} \begin{aligned} \kappa_{\theta^{'}\theta^{'}}=\kappa(r,\theta)\frac{R_{2}-r}{R_{2}-\frac{R_{1}}{5}\left( \sin\left( 3\theta\right)+2 \right)}\left( \frac{R_{2}-\frac{R_{1}}{5}\left( \sin\left( 3\theta\right)+2 \right)}{R_{2}-R_{1}}\frac{\left( r-R_{1} \right)}{r}+\frac{R_{1}\left( \sin\left( 3\theta\right)+2 \right)}{5r} \right)\# \end{aligned}\#\left( S50c \right) \end{aligned}$$

where

$$\alpha_{1}=\frac{R_{2}-\frac{R_{1}}{5}\left( \sin\left( 3\theta\right)+2 \right)}{{(R}_{2}-R_{1})\left( \frac{R_{2}-\frac{R_{1}}{5}\left( \sin\left( 3\theta\right)+2 \right)}{R_{2}-R_{1}}\left( r-R_{1} \right)+\frac{R_{1}}{5}\left( \sin\left( 3\theta\right)+2 \right) \right)}$$

$$\alpha_{2}=\frac{9\left( R_{2}-r \right)^{2}R_{1}^{2}\cos^{2} \left( 3\theta\right)}{25\left( R_{2}-R_{1} \right)\left( R_{2}-\frac{R_{1}}{5}\left( \sin\left( 3\theta\right)+2 \right) \right)}$$

$$\alpha_{3}=\frac{1}{\left( \frac{R_{2}-\frac{R_{1}}{5}\left( \sin\left( 3\theta\right)+2 \right)}{R_{2}-R_{1}}\left( r-R_{1} \right)+\frac{R_{1}}{5}\left( \sin\left( 3\theta\right)+2 \right) \right)}\frac{1}{r}$$

Similarly, when $r<r_{1}$, the thermal conductivity is set to $\kappa$ = $\kappa_{0}$. The resulting thermal conductivity distribution is shown in Figure S4**c**.

**S3: Supplementary analysis of temperature profiles and heat flux distribution in active-source camouflage.**

In the main text, we have demonstrated both simulation and experimental results showing the successful camouflage of a circular active source into various shapes. Here, we provide supplementary details regarding the temperature and heat flux profiles associated with these transformations. As shown in Figure S5**a**, the heat flux direction for the circular active source, camouflaged into triangular, square, and clover shapes, exhibits a radial outward flow. Figure S6**b** presents the temperature profile along the vertical central axis for the circular active source camouflaged into a clover shape, with the temperature controlled within the range of 350-1000K. Figure S6**c** further illustrates the corresponding anisotropic thermal conductivity distribution. Figs. S7**a** and S7**b** confirm that the heat flux maintains its radial flow, with the distribution remaining invariant even as the temperature increases. Figs. S8**a**, S8**b**, and S8c provide additional insights into the heat flux direction and temperature profiles for active sources camouflaged into two-leaf clover, four-point star, and five-leaf shapes, respectively. These figures also display the heat flux distribution at key boundaries, including those of the camouflaged active source, the original source, and the design region. Finally, Figs. S9-S11 present the thermal conductivity distribution maps corresponding to the results shown in Figure 3**g** of the main text. Together, these supplementary results substantiate the effectiveness of our ASM design in achieving precise and robust thermal management for active-source thermal fields, underscoring the versatility and reliability of our approach in diverse thermal environments.

**S4: Detailed analysis of active-source camouflage under varying geometries and power levels.**

In the following, we provide further insights into the camouflage of active sources with arbitrary shapes and power levels. As illustrated in Figure S12, the heat flux exhibits radial flow, and the corresponding temperature profile along the central axis confirms that, despite achieving the camouflage effect, the temperature field in the background region remains unaffected. Subsequently, we present the thermal conductivity distribution, which reveals that larger original active-source shapes correspond to higher thermal conductivity values, as shown in Figure S13 and S14. Additionally, we conduct an analysis of heat flux and thermal conductivity distributions under varying power conditions. In Figure S15, the heat flux values at specific radial positions (r = 0.05 m, r = 0.08 m, and r = 0.1 m) on a circle at θ = 60° are plotted for different power levels, with the blue, green, and yellow lines representing different power settings. It is observed that the heat flux increases linearly with power, while the thermal camouflage effect remains invariant. Furthermore, Figure S16 and S17 show the relationship between the reciprocal of thermal conductivity and active-source power, revealing an inverse proportionality between them. This indicates that as the power of the active source increases, the thermal conductivity also increases. Collectively, these results substantiate the robustness and general applicability of our ASM design theory, confirming its efficacy in achieving precise thermal field management across various source geometries and power conditions.

**S5: Designing ASM for anisotropic thermal conductivity via topology optimization.**

Since materials with anisotropic thermal conductivity are nearly non-existent in nature, achieving equivalent anisotropic thermal conductivity in ASM is crucial. Inspired by the work of *Qiu et al*., we aim to fabricate ASM that exhibit equivalent anisotropic thermal conductivity using a single natural material^[7,8]^. According to Fourier's law, the heat flux density is $J_{T}=-\kappa\nabla T$, and the heat flux through a heat transfer channel with area A is $q=-\kappa A\nabla T$. This implies that it is feasible to control the heat flux field and temperature field by modifying either the thermal conductivity or the area of the heat transfer channel.

Here, we first divide the thermal conductivity into ten layers following traditional methods, determining the isotropic thermal conductivity for each layer through topology optimization to achieve effective anisotropic thermal conductivity. We then translate each layer’s isotropic thermal conductivity into the corresponding size of the heat channel area. This process ultimately results in the structural design of the experimental setup (Figure S18**a)**. The resulting experimental panel is shown in Figure S18**b** and **c**, with the layer structure detailed in Table S1. To minimize the impact of convection on the panel's functionality, a convection coefficient of h=10 W$\cdot m^{-2}\cdot K^{-1}$ is specified during the topology optimization process. Additionally, to achieve a more uniform temperature distribution in the camouflaged active source at the center, the height of the high thermal conductivity region in the panel's center is described by $z=c+ar^{2}$, where c=0.00714, a=8, and $r$ is the distance from the center. The thickness of the panel in the background region is 2.5 mm. This design method effectively avoids issues such as thermal contact resistance inherent in traditional composite materials while preserving the mechanical properties of the ASM.

**S6: Validation of ASM functionality through numerical simulations.**

Next, we validate the functionality of these structures through numerical simulations, as shown in Figure S19. In these simulations, the material's thermal conductivity is set to 176$W\cdot m^{-2}\cdot K^{-1}$, the bottom active source power is set to 10000 W$\cdot m^{-2}$, and the boundary temperature is set to 293 K. The results clearly demonstrate the successful camouflage of the circular active source into triangular, square, and cloverleaf shapes, as well as the ability to control the temperature within the active-source region.

Moreover, fabrication inaccuracies may lead to variations in the thickness of different layers, which could potentially affect the thermal conductivity distribution and, consequently, the camouflage effect. The metal 3D printing process used in our fabrication has an accuracy of 0.05 mm, while the thinnest section of our experimental panel is 2 mm. This suggests that fabrication deviations are relatively small compared to the overall thickness and are unlikely to significantly affect the camouflage performance. To further assess the impact of fabrication errors, we conducted additional numerical simulations in which the thickness of the entire experimental panel was uniformly increased or decreased by 0.05 mm across all regions. The simulation results (Figure S20) showed that the thermal camouflage effect remained nearly unchanged, indicating the robustness of our approach. However, we observed that the temperature of the active source increased or decreased by approximately 2 K, suggesting a minor effect on the temperature but no significant degradation of the camouflage functionality. Furthermore, to evaluate the impact of interfacial thermal resistance, we consider two extreme scenarios: PDMS being completely non-conductive and PDMS fully conducting heat. In both cases, the effect can be equivalently represented as an increase or decrease of approximately 0.1 mm in the thickness of the experimental panel (Figure S21). Therefore, the impact of interfacial thermal resistance can be considered negligible.

**S7: Experimental system as well as presentation of transient results**

Here, we construct the experimental setup, with the corresponding actual device shown in Figure S22. To ensure uniform emissivity across the surface of the experimental panel, we apply a thin, uniform layer of black ink on its surface. The experimental panel is connected to a water bath at 293 K via an external water trough to establish a low-temperature boundary. We connect the electrical circuit between the voltage source and the circular heating element, and use a digital ammeter to measure the circuit current, which is 0.3 A, and the voltage, which is 170 V. To minimize thermal resistance, a thin layer of thermal grease is applied between the experimental panel and the active source. After cooling the experimental panel to room temperature, the experiment is initiated, and thermal imaging is recorded using an infrared thermal camera.

Our observations reveal that the designed structure exhibits transient characteristics, with the thermal camouflage pattern consistently appearing from the start to the end of the experiment. Over time, the temperature of the camouflaged pattern at the center increases. The time required to reach steady-state is approximately 200 seconds for each experimental setup, as shown in Figure S23**a**. Compared to traditional thermal metamaterials, our experimental setup demonstrates a very fast thermal response. Subsequently, a line graph analysis of the temperature along the central axis of the transient image is conducted, as shown in Figure S23**b**. It can be observed that the temperature within the camouflaged active source region reaches its peak, approaching 400 K, while the thermal fields in the surrounding region remains undisturbed.

Additionally, it is clearly observed that higher temperatures in the design process resulted in faster temperature rises during the transient phase, as depicted in Figure S24**a**. Furthermore, no distortions are observed in the external background area in any of our experiments, highlighting the potential of this method for the development of thermal metamaterials with transient functionalities, as shown in Figure S24**b**.

**S8:** **More generalized simulation results of active-source thermal camouflage.**

In Figure S25, we present a set of simulation results demonstrating thermal camouflage for highly arbitrary-shaped active sources. The results clearly show that any original active source can be effectively camouflaged into any desired shape. These simulations strongly validate the capability of our proposed ASM in achieving efficient and versatile thermal camouflage for arbitrary heat sources.

**S9: Stability assessment of the ASM.**

Figure S26 presents the thermal conductivity measurements of the ASM material, obtained using a laser flash analyzer over 100 heating cycles ranging from 293 K to 450 K. The results exhibit no significant variation in thermal conductivity throughout the repeated thermal cycling, providing strong evidence for the robustness and long-term stability of the ASM material.


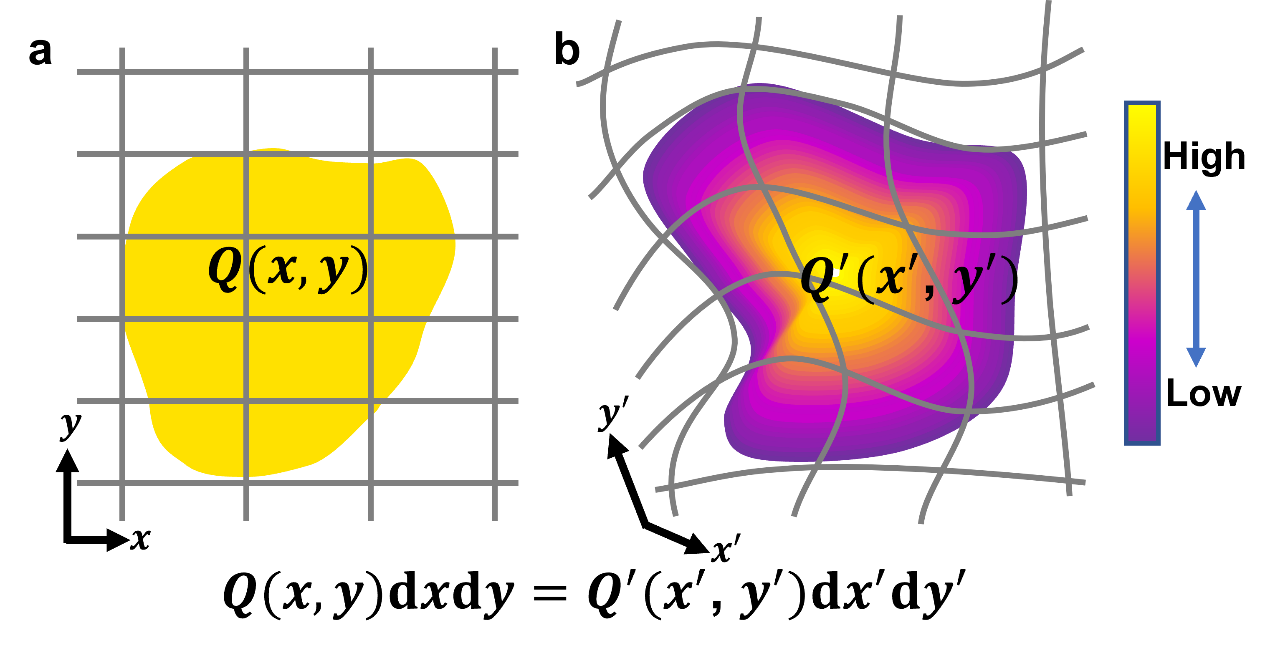


**Figure S1:** Illustration of the transformation relationship of the active source. **a** represents the uniform active source in the original coordinate system, while **b** represents the corresponding active source in the transformed coordinate system. They satisfy the law of energy conservation, meaning the heat generation per unit area is the same.


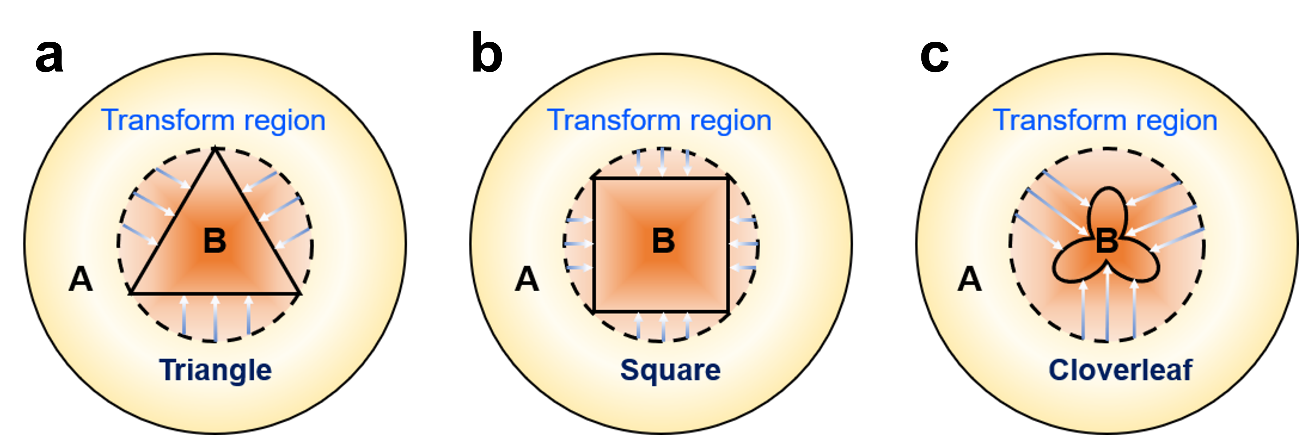


**Figure S2:** Schematic diagram of the design principles for camouflaging a circular active source. **a** shows the radial compression transformation of a circle into a triangular shape. **b** shows the radial compression transformation of a circle into a square shape. **c** shows the radial compression transformation of a circle into a cloverleaf shape.


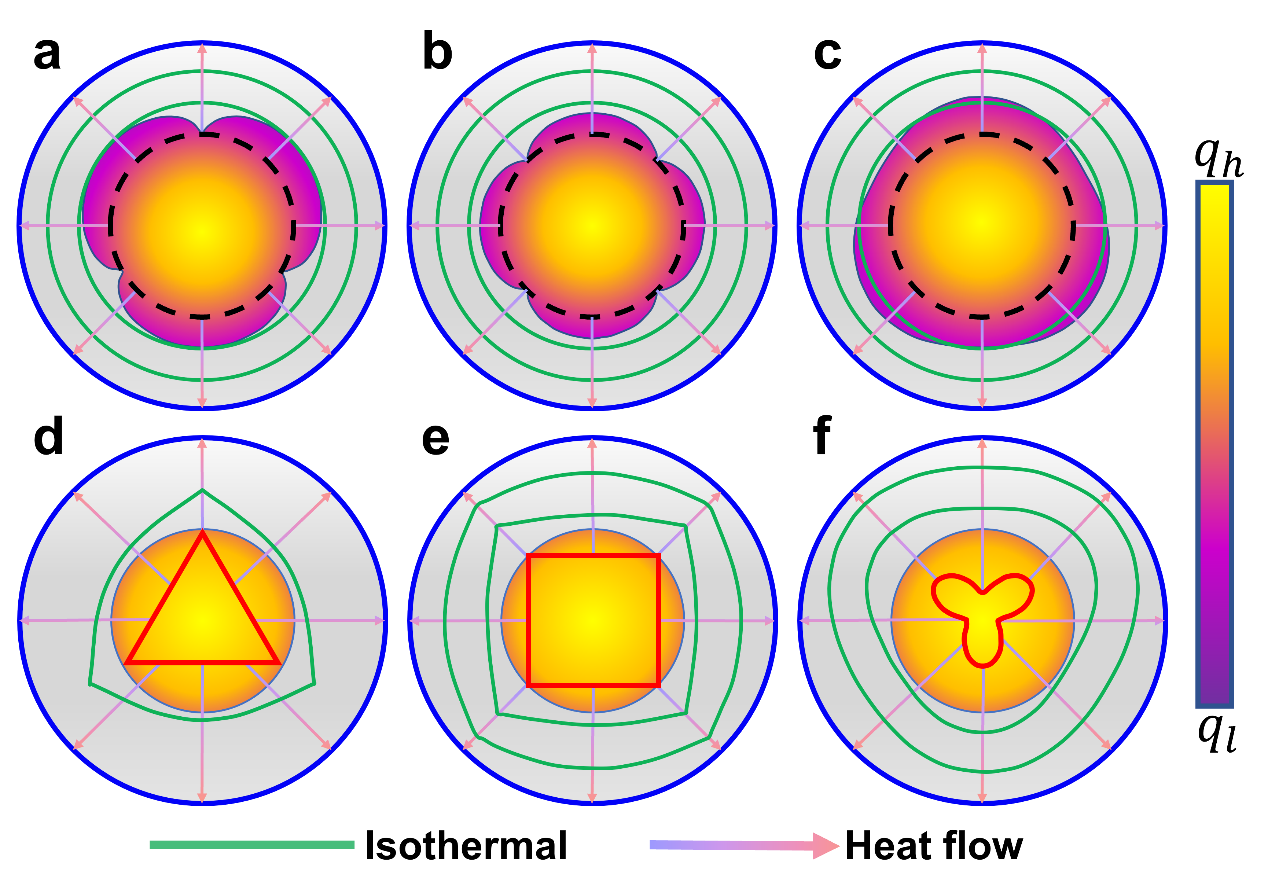


**Figure S3:** Schematic diagram illustrating temperature and heat flux fields in the predefined and target thermal fields. **a**, **b**, and **c** depict the temperature and heat flux distributions within the predefined thermal fields, where green lines denote isotherms and purple arrows indicate heat flux directions. The central yellow-purple region denotes the power and position of the circular active source in the target thermal fields relative to its configuration in the predefined thermal fields. **d**, **e**, and **f** illustrate the active source, temperature field, and heat flux distribution within the target thermal fields.


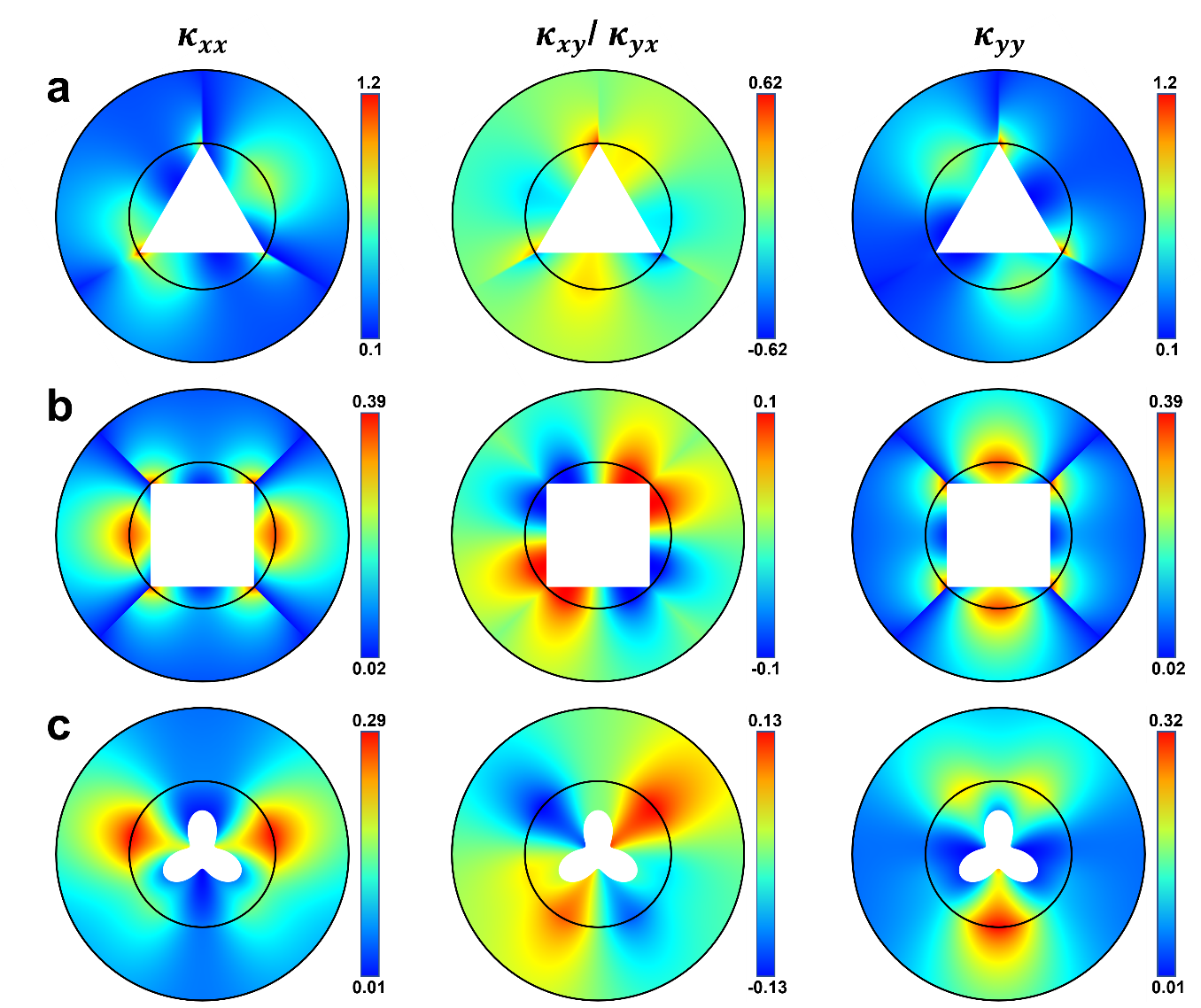


**Figure S4:** Anisotropic thermal conductivity distribution: **a**, **b**, and **c** show the thermal conductivity distribution of the circular active source camouflaged into triangular, square, and clover-shaped profiles, respectively. The central white region represents an isotropic high thermal conductivity area.


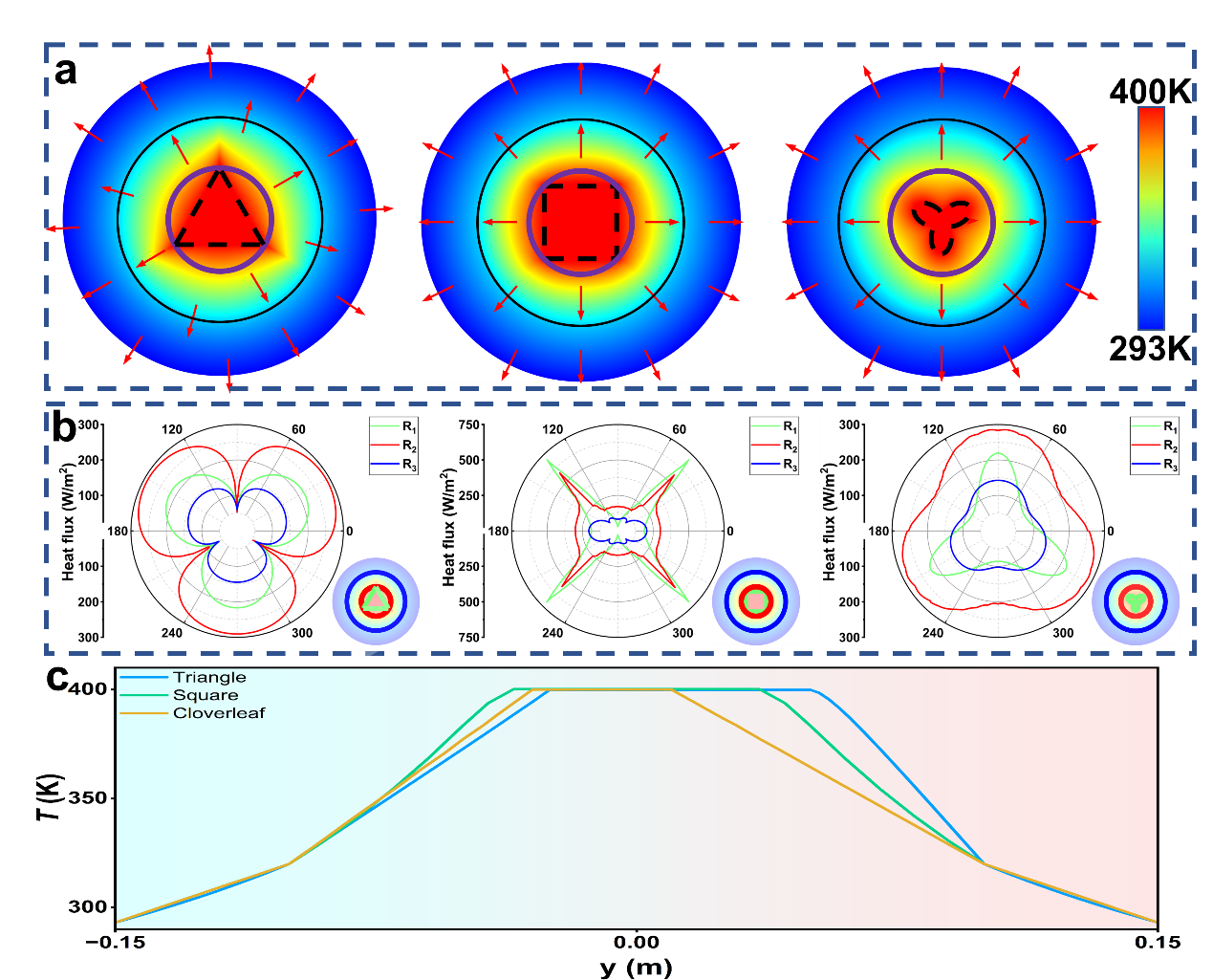


**Figure S5:** Supplementary explanation for Figure 2: **a** The direction of heat flux flows radially, **b** Heat flux distribution, and **c** Temperature analysis along the central axis.


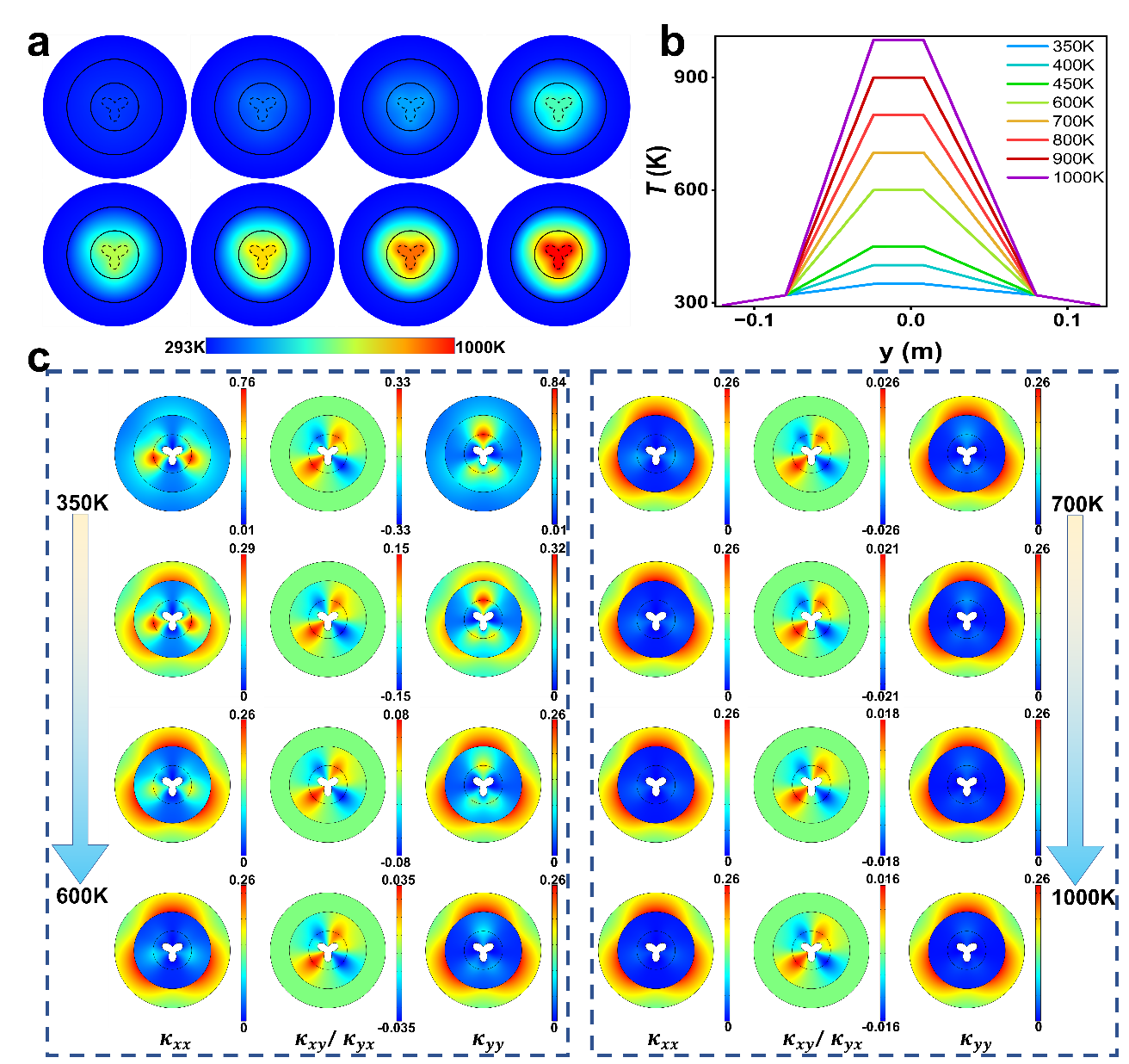


**Figure S6:** Supplementary information on temperature and anisotropic thermal conductivity for Figure 3: **a** Camouflaging the circular active source into a clover shape and controlling the temperature range of the active source from 350K to 1000K; **b** Temperature profile along the vertical central axis; **c** Anisotropic thermal conductivity distributions.


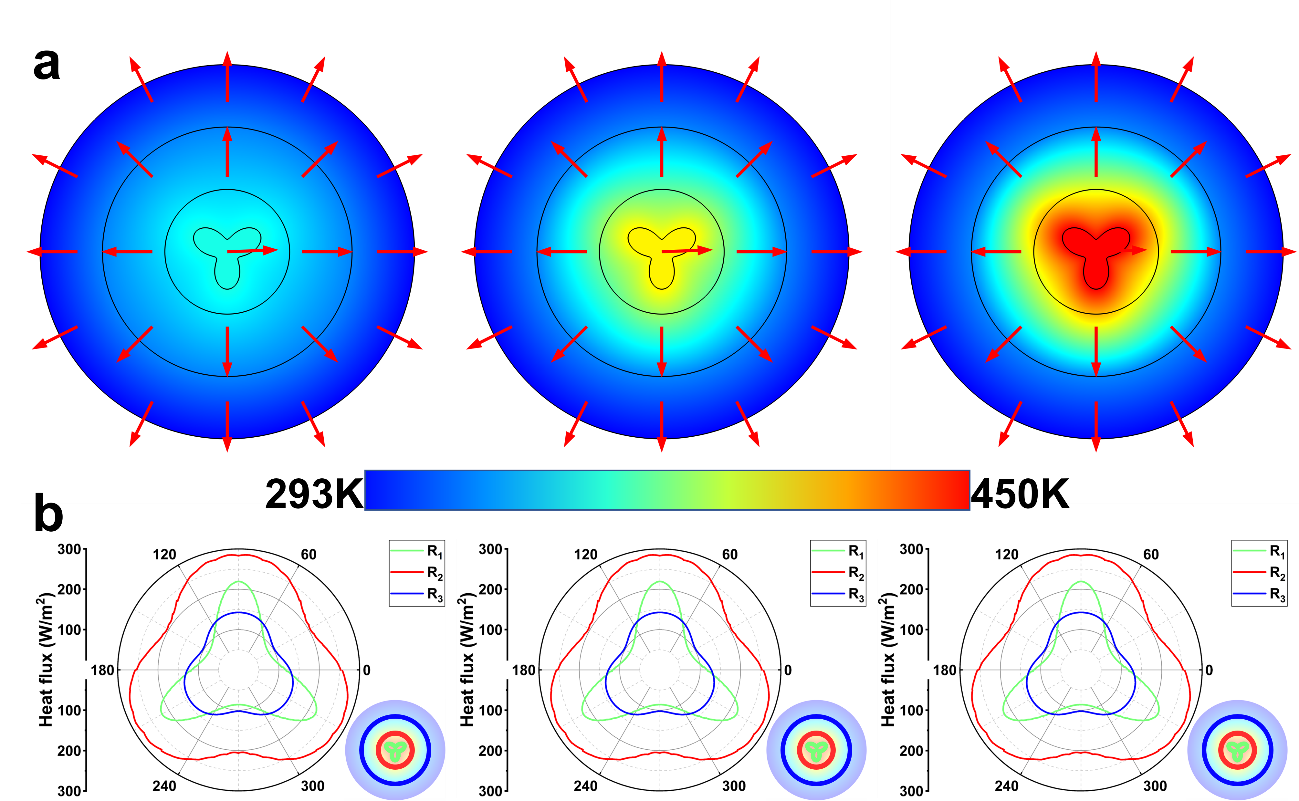


**Figure S7:** Supplementary information on heat flux direction and magnitude for Figure 3: **a** The heat flux direction flows radially outward; **b** Heat flux analysis at the boundaries of the camouflaged clover-shaped active source, the original circular active source, and the transformation region. It can be observed that while the temperature increases, the heat flux remains unchanged.


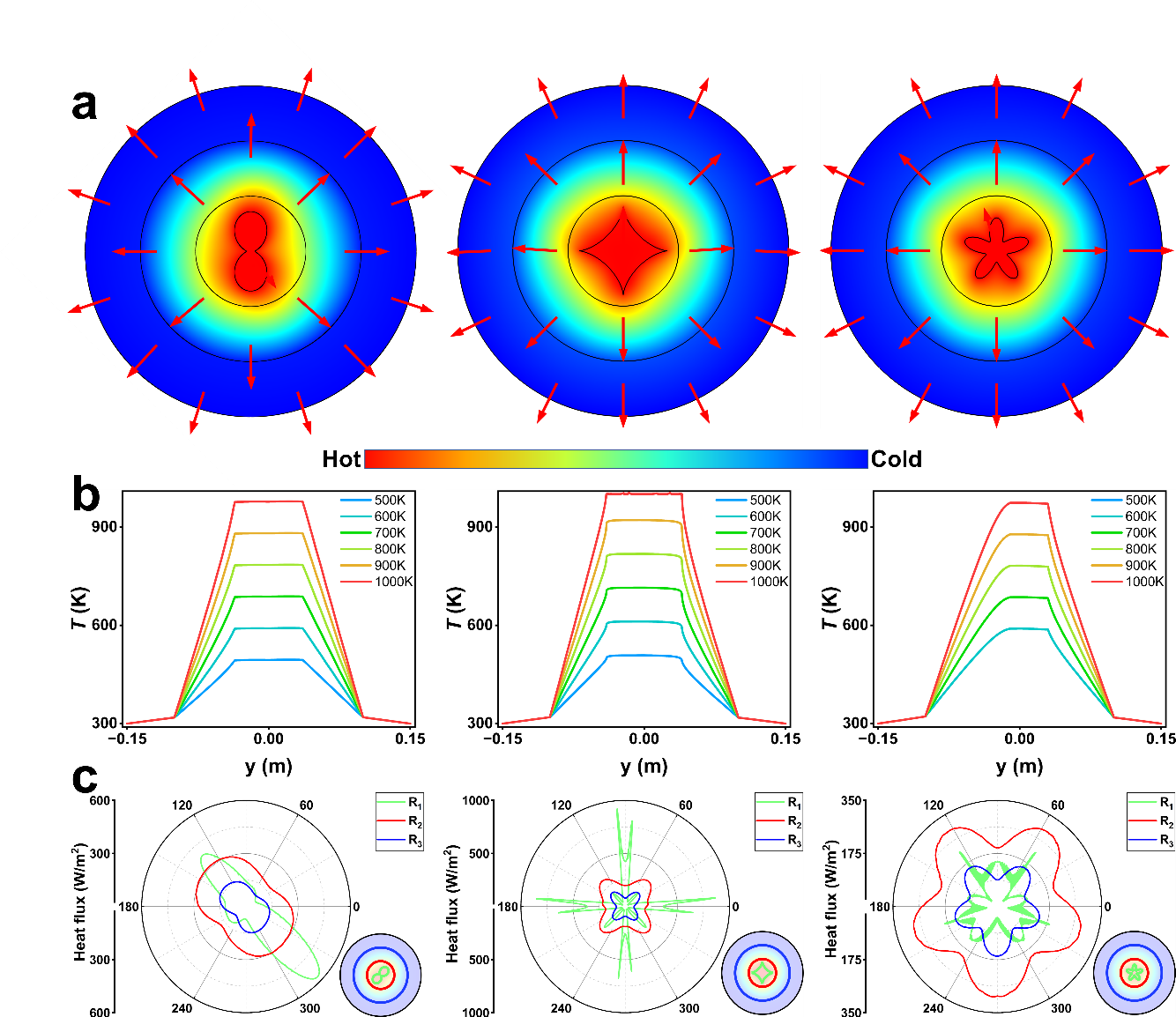


**Figure S8:** Heat flux and temperature analysis for camouflaging a circular active source into cloverleaf, four-point star, and five-leaf shapes: **a** The heat flux direction flows radially outward; **b** Temperature profile along the vertical central axis; **c** Heat flux analysis at the boundaries of the camouflaged active source, the original active source, and the transformation region.


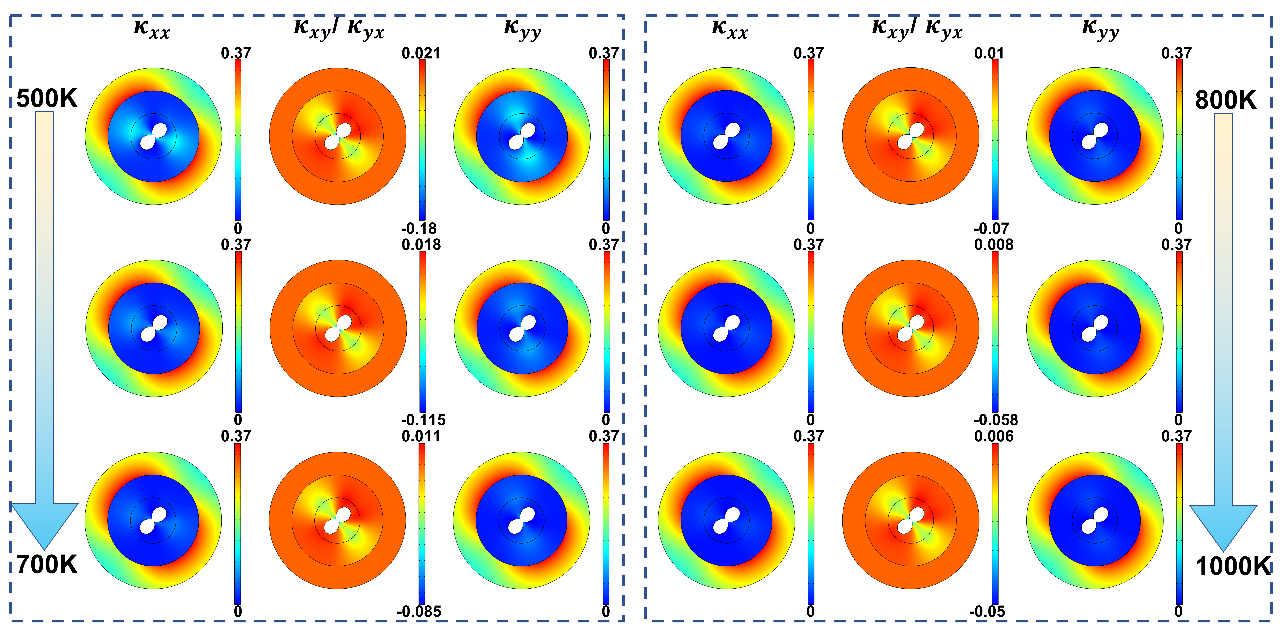


**Figure S9:** Anisotropic thermal conductivity distribution map for camouflaging a circular active source into a two-leaf shape while controlling the active-source temperature from 500K to 1000K.


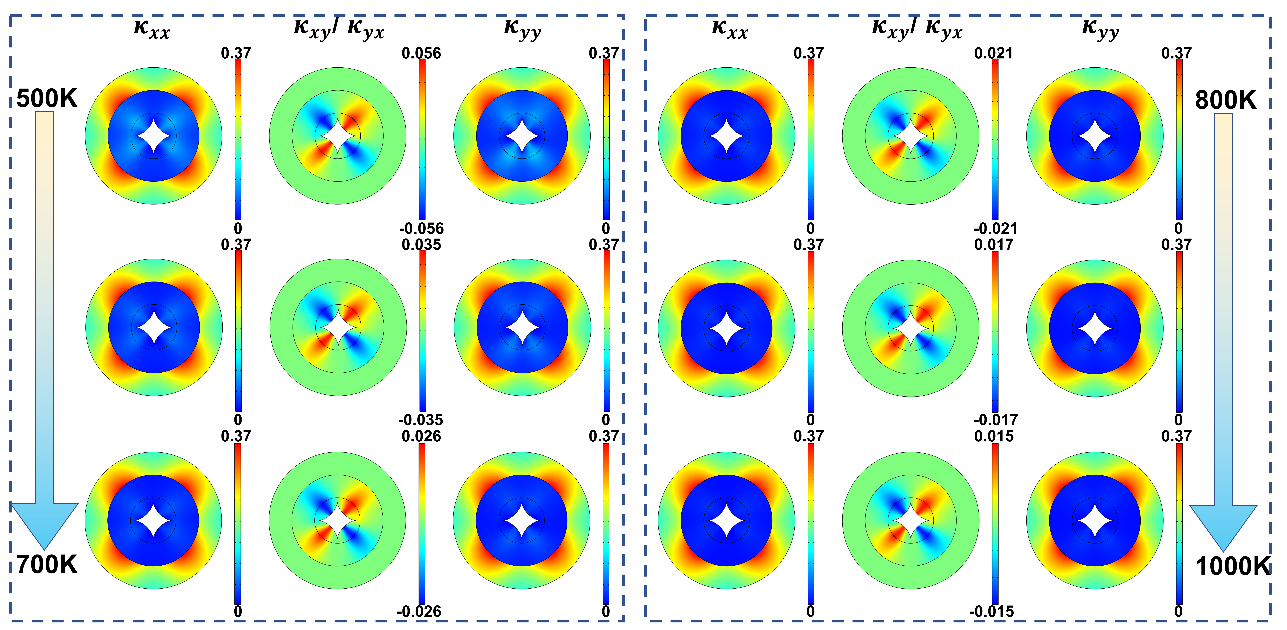


**Figure S10:** Anisotropic thermal conductivity distribution map for camouflaging a circular active source into a four-point star shape while controlling the active-source temperature from 500K to 1000K.


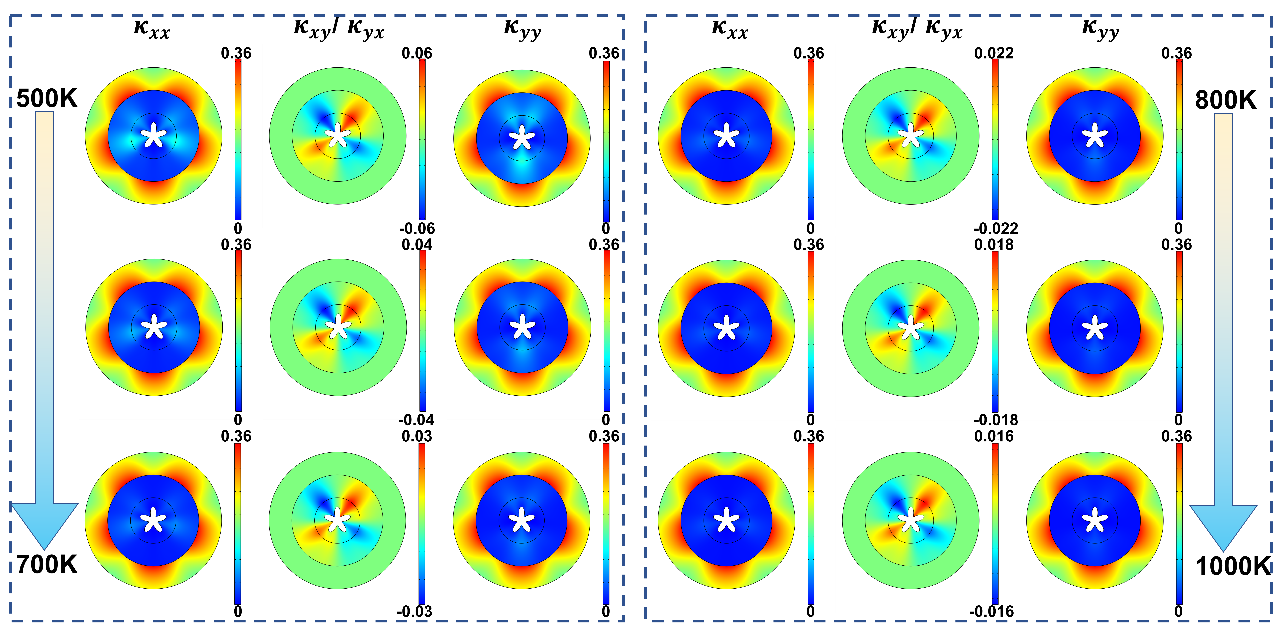


**Figure S11:** Anisotropic thermal conductivity distribution map for camouflaging a circular active source into a five-leaf shape while controlling the active-source temperature from 500K to 1000K.


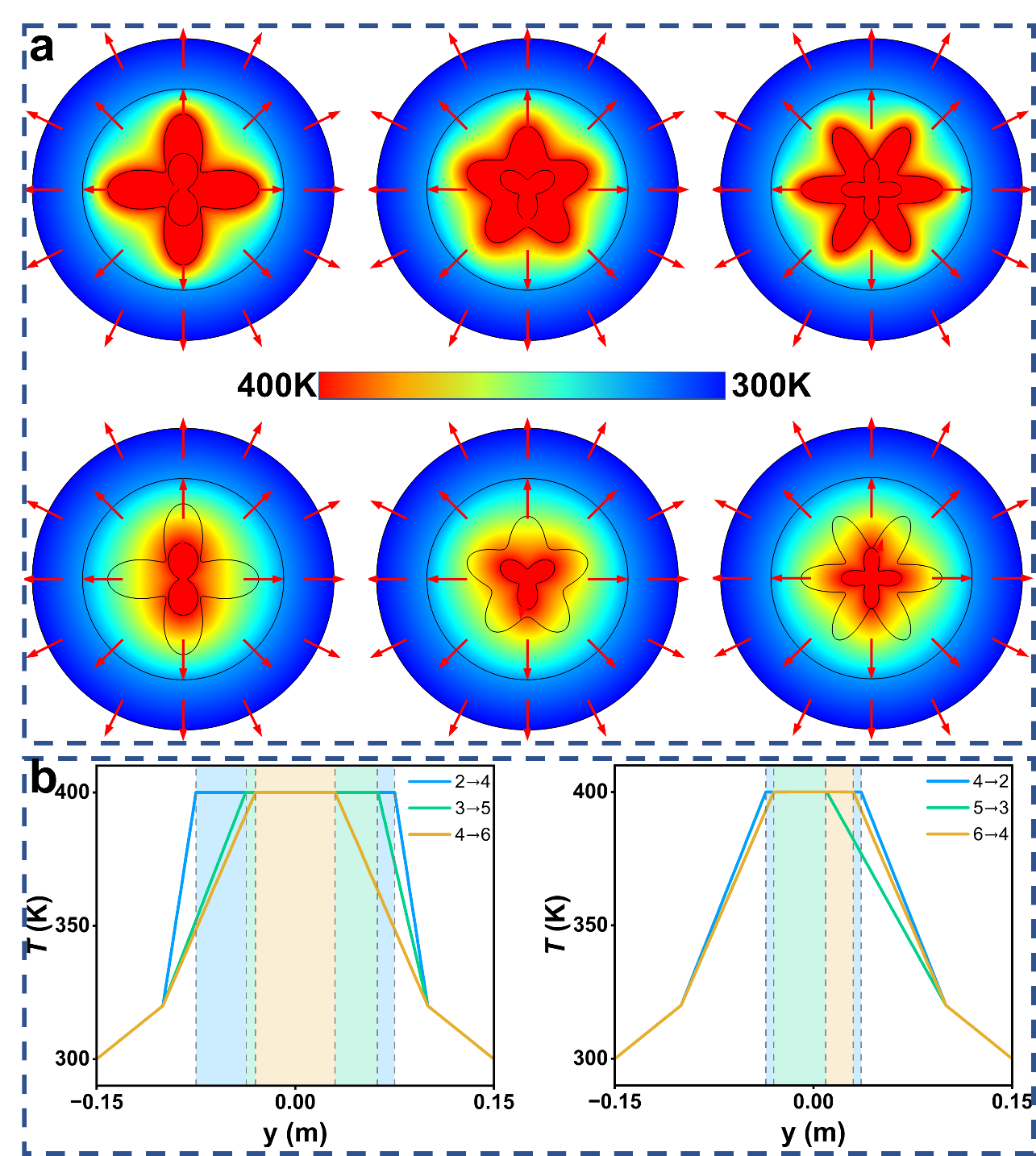


**Figure S12:** Supplementary explanation of heat flux and temperature for Figure 4: **a** Heat flux flows radially outward; **b** Temperature profile along the vertical central axis.


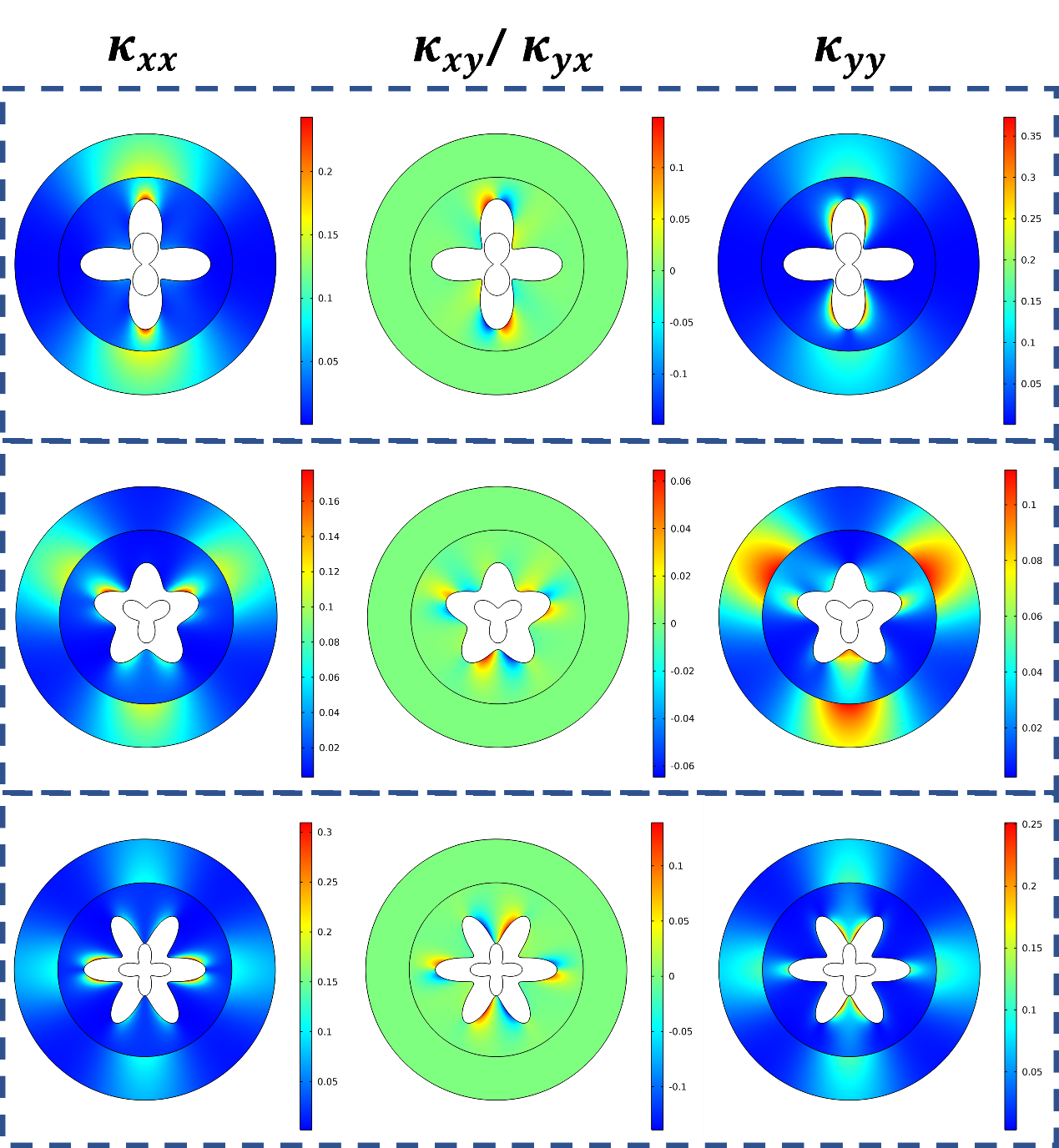


**Figure S13:** The thermal conductivity distribution maps of camouflaging two-leaf, three-leaf, and four-leaf active sources into four-leaf, five-leaf, and six-leaf shapes.


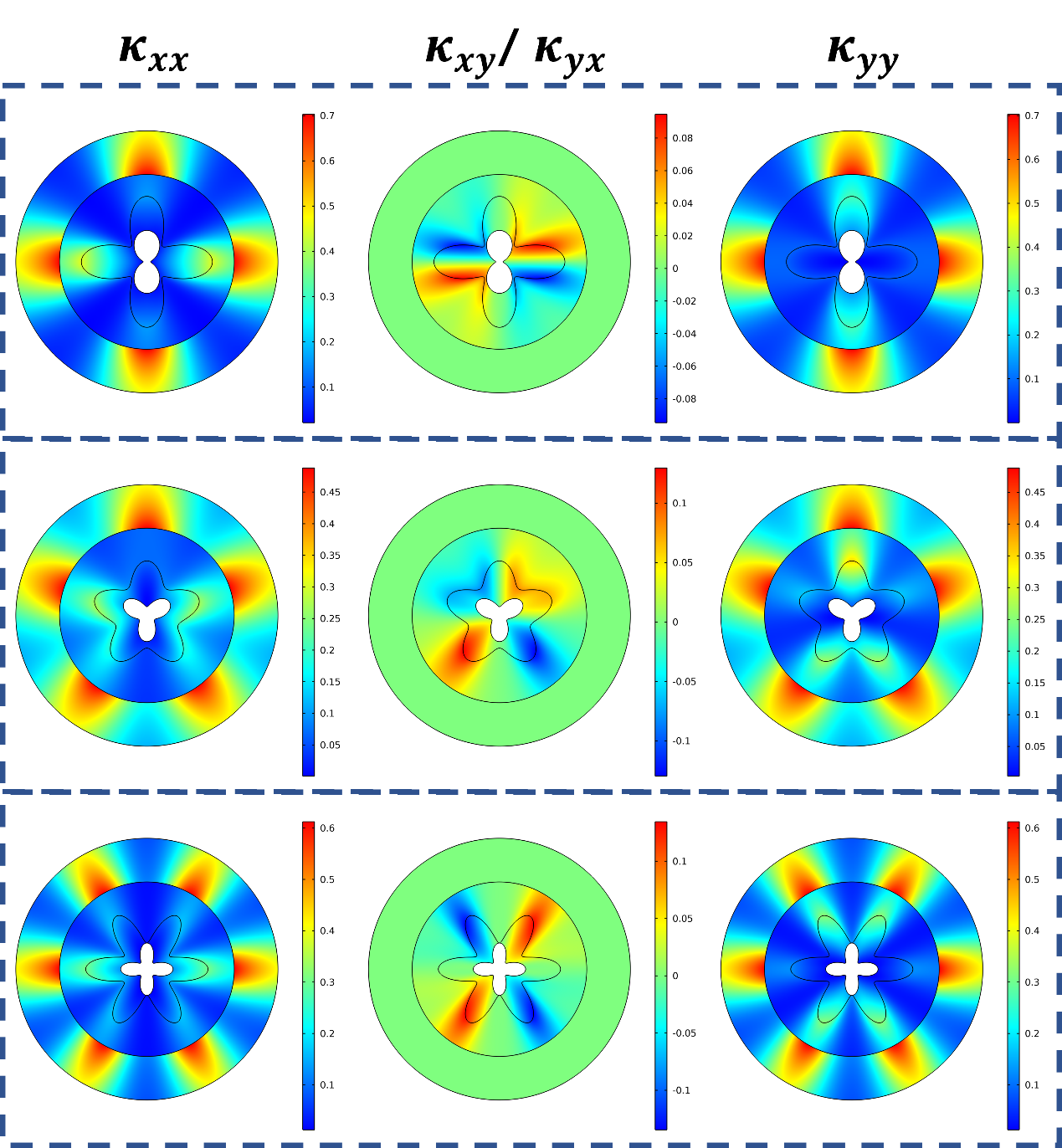


**Figure S14:** The thermal conductivity distribution maps of camouflaging four-leaf, five-leaf, and six-leaf active sources into two-leaf, three-leaf, and four-leaf shapes.


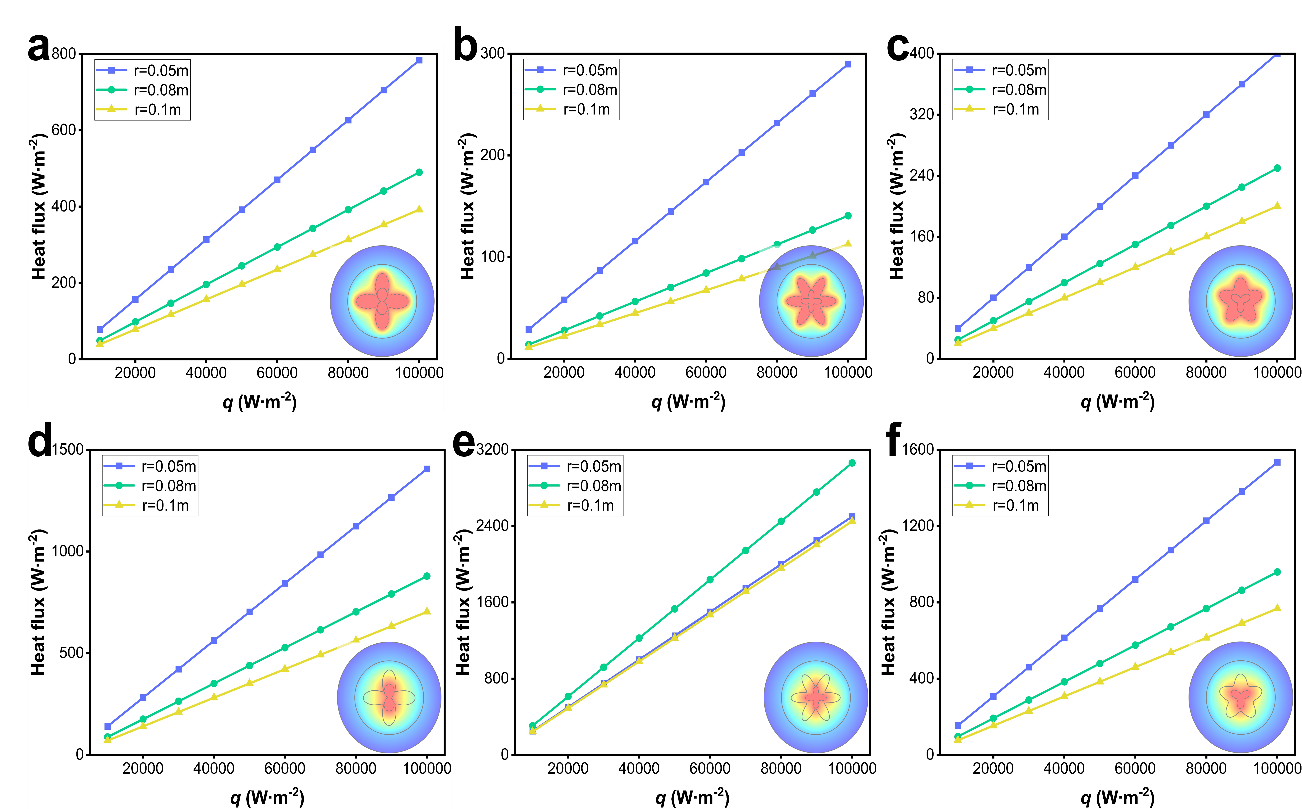


**Figure S15:** Supplementary analysis of achieving the same thermal camouflaging functionality under different active-source powers. **a-f** Present the analysis of heat flux magnitude under varying power levels while maintaining the same thermal camouflaging functionality. The blue, green, and yellow lines represent the heat flux values at a point on the circle with $r=0.05 m$, $r=0.08 m$, $r=0.1 m$, and $\theta$= 60° under different power levels.


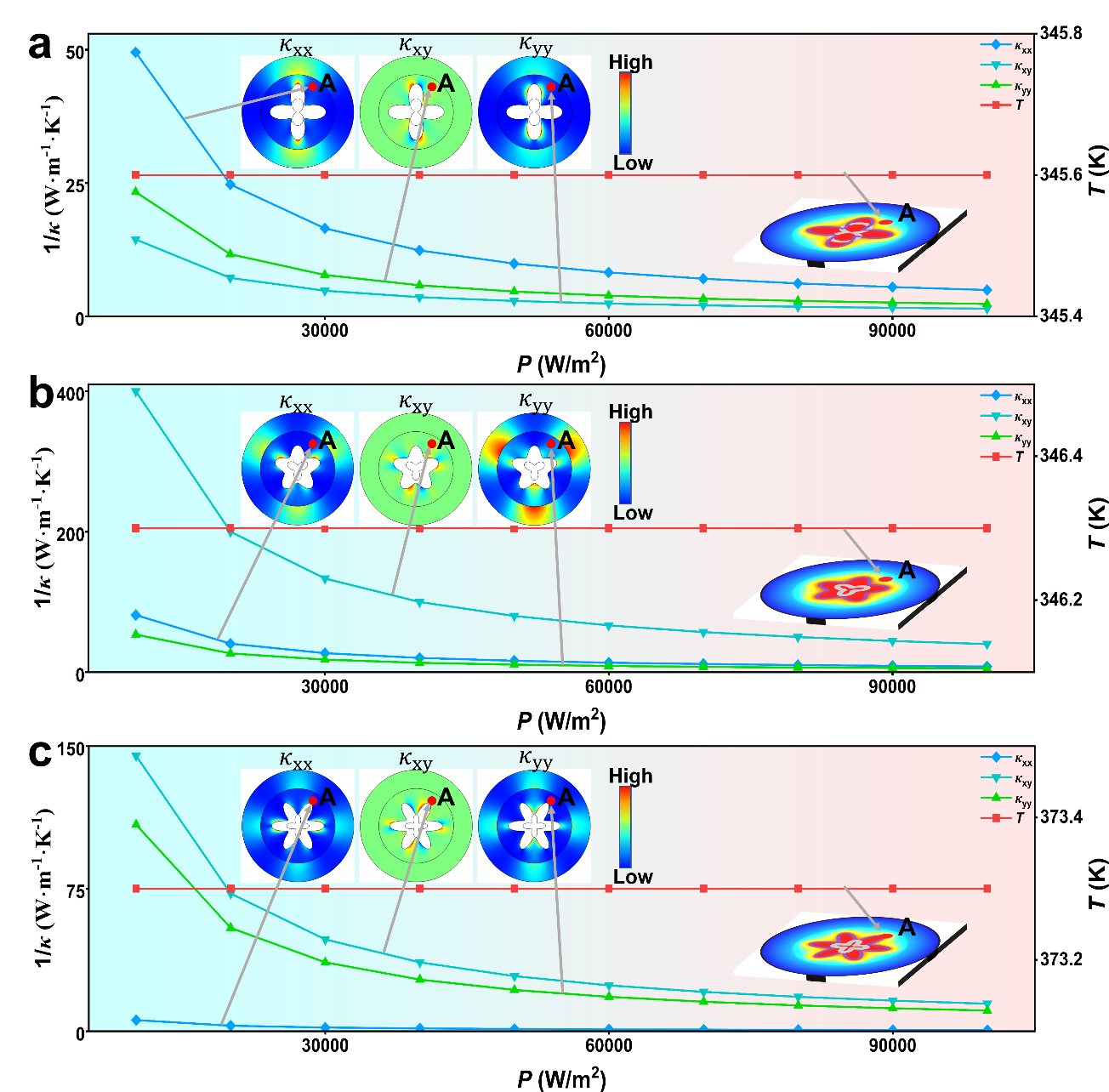


**Figure S16:** Figures **a**, **b**, and **c** illustrate the inverse thermal conductivity at a specific point (r=0.08 m, $\theta$=60°) for active sources undergoing camouflage transformations from two-leaf, three-leaf, and four-leaf shapes into four-leaf, five-leaf, and six-leaf configurations, respectively. The relationship between the inverse thermal conductivity and active-source power (ranging from 10,000 W to 100,000 W) is systematically analyzed.


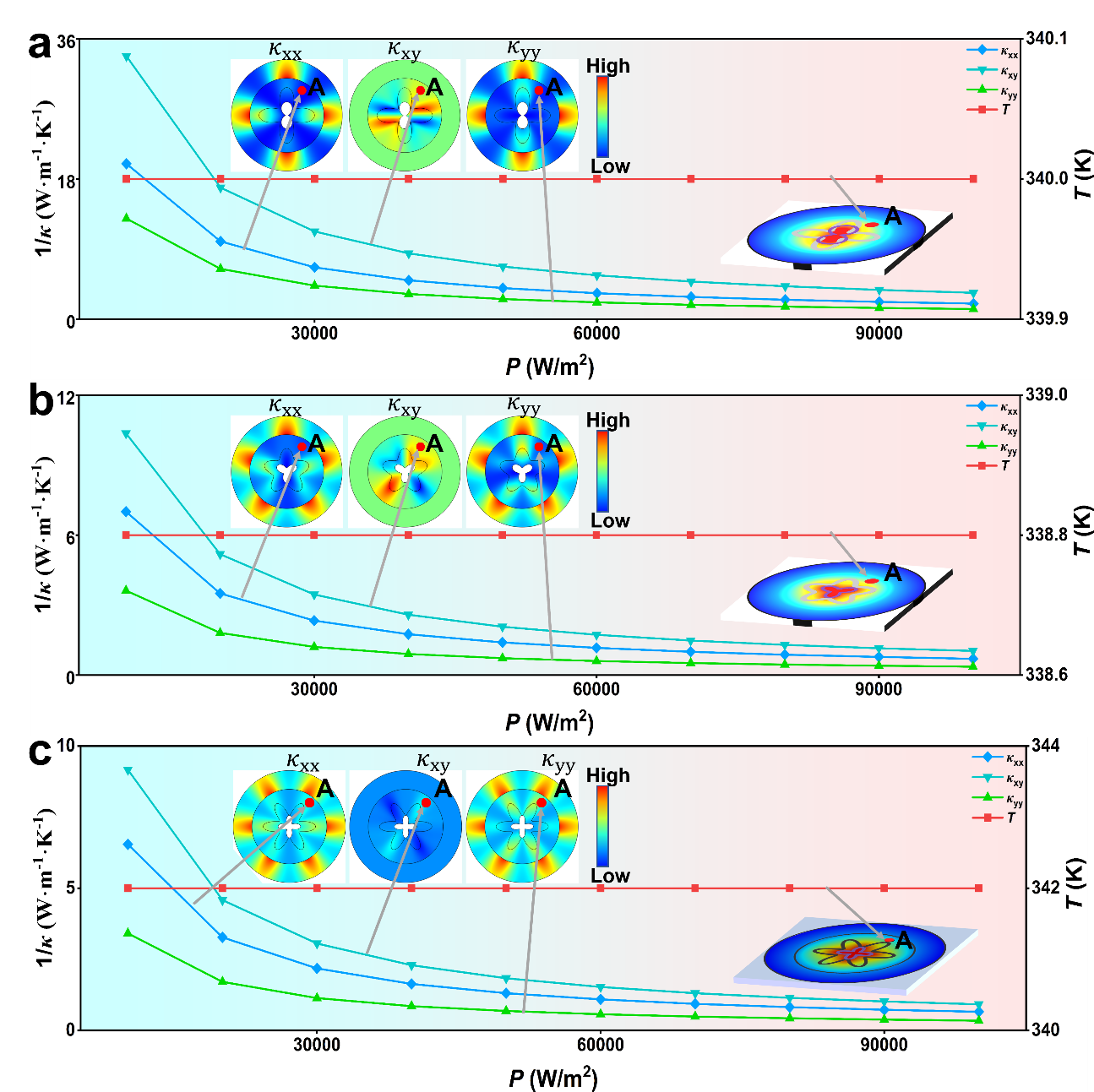


**Figure S17:** Figures **a**, **b**, and **c** respectively represent the inverse thermal conductivity at a specific point (r=0.08 m, $\theta$=60°) for active sources camouflaged from two-leaf, three-leaf, and four-leaf shapes into four-leaf, five-leaf, and six-leaf configurations. These graphs show the functional relationship between the inverse thermal conductivity and the active-source power, ranging from 10,000 W to 100,000 W.


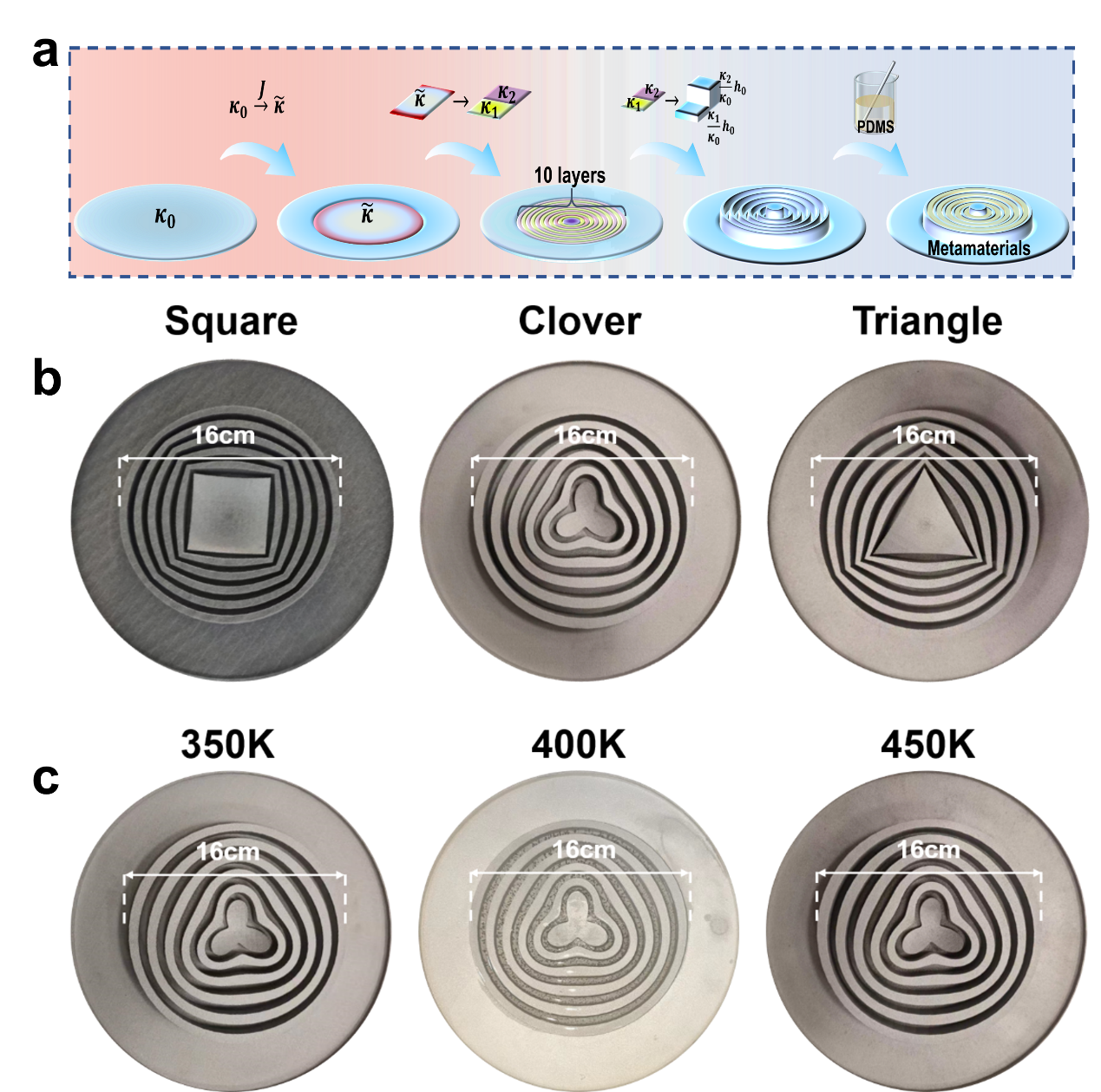


**Figure S18:** Schematic diagram and photographs of the experimental structure. **a** illustrates the principle of the equivalent thermal conductivity design. **b** shows photographs of the experimental panels where the circular active source is camouflaged into square, cloverleaf, and triangular shapes. **c** presents the experimental panels with the active-source temperatures regulated to 350 K, 400 K, and 450 K, respectively.


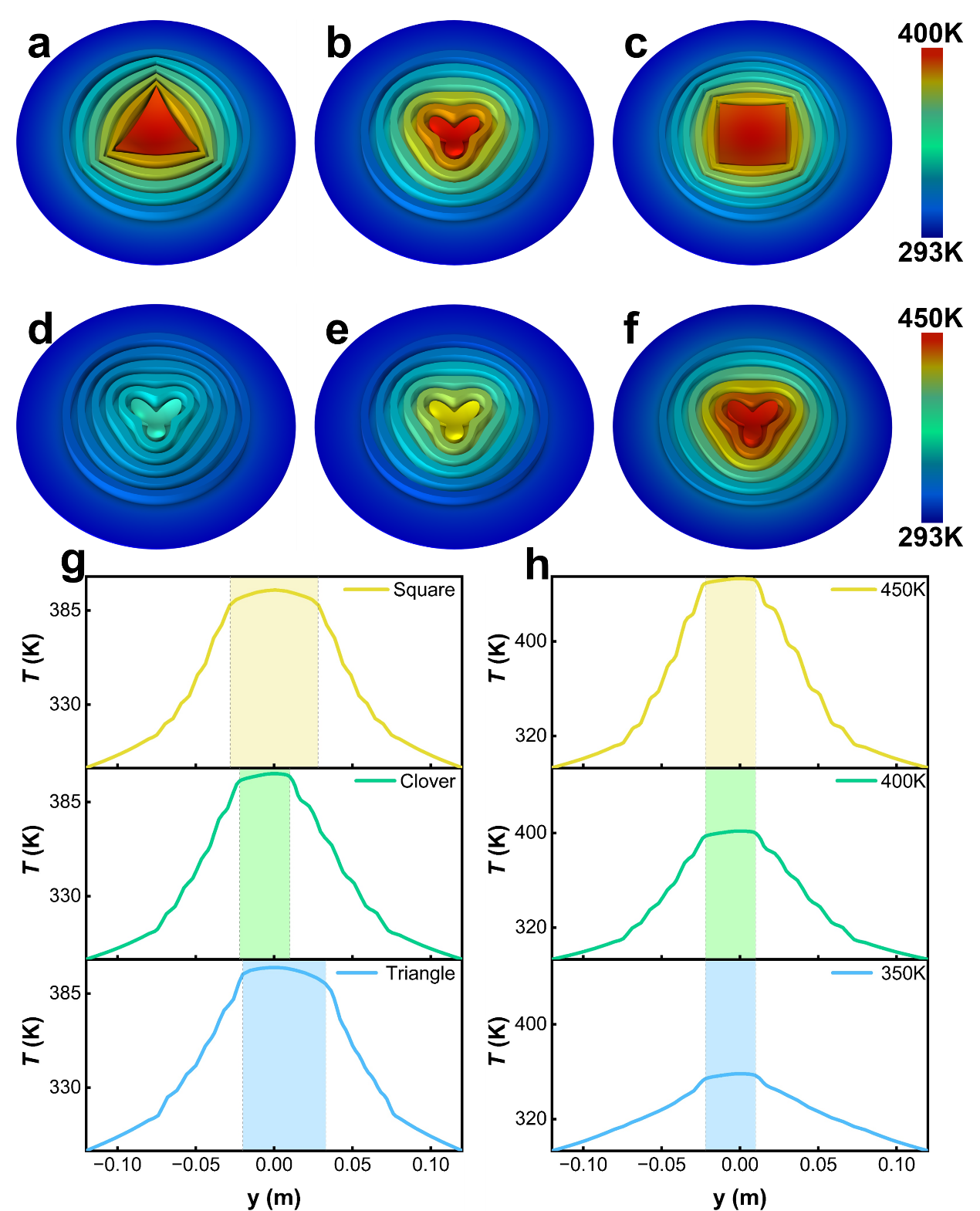


**Figure S19:** Simulation results of the experimental panel. **a-f** show simulations of the experimental panel with a active-source power of $10000 W m^{-2}$, a convection heat transfer coefficient of 10 W·$m^{-2}$·$K^{-1}$, and a boundary temperature condition of 293 K. **g** and **h** depict the temperature values along the vertical central axis, with the shaded regions representing the camouflaged active-source areas.


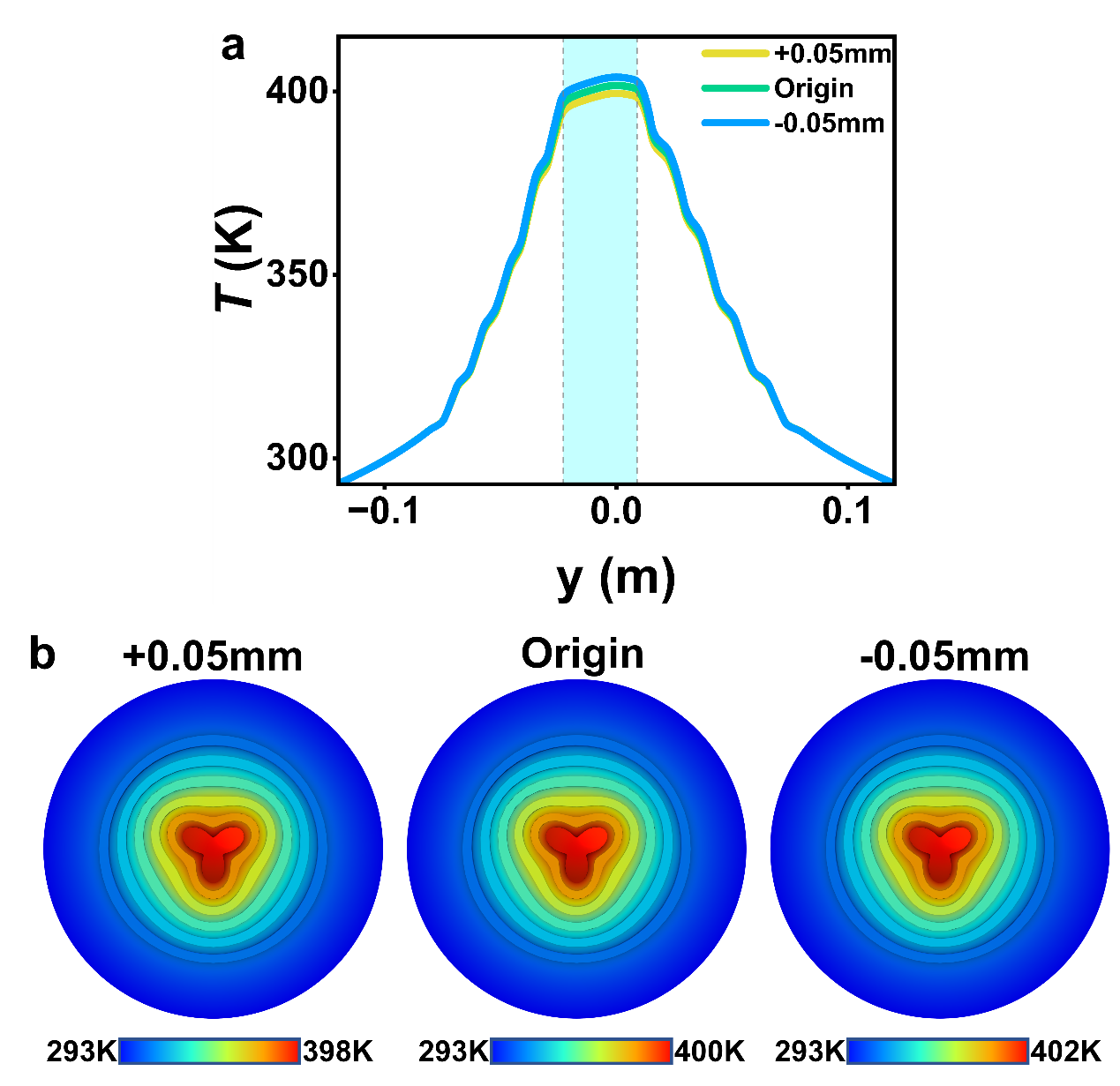


**Figure S20.** **a** Temperature profile analysis along the vertical central axis. **b** Simulation results of the 3D experimental panel with a ±0.05 mm thickness variation.


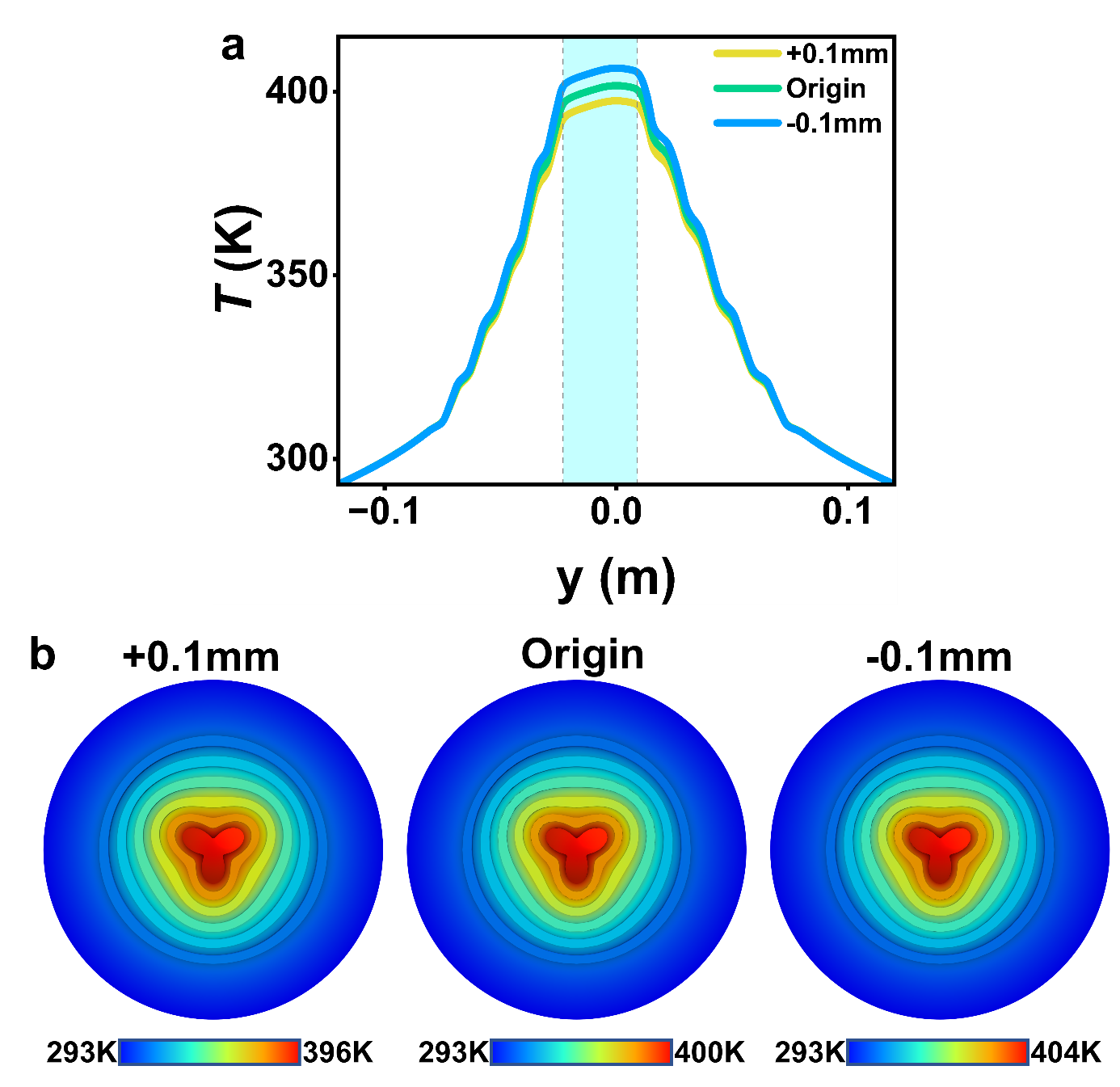


**Figure S21.** **a** Temperature profile analysis along the vertical central axis. **b** Simulation results of the 3D experimental panel with a ±0.1 mm thickness variation.


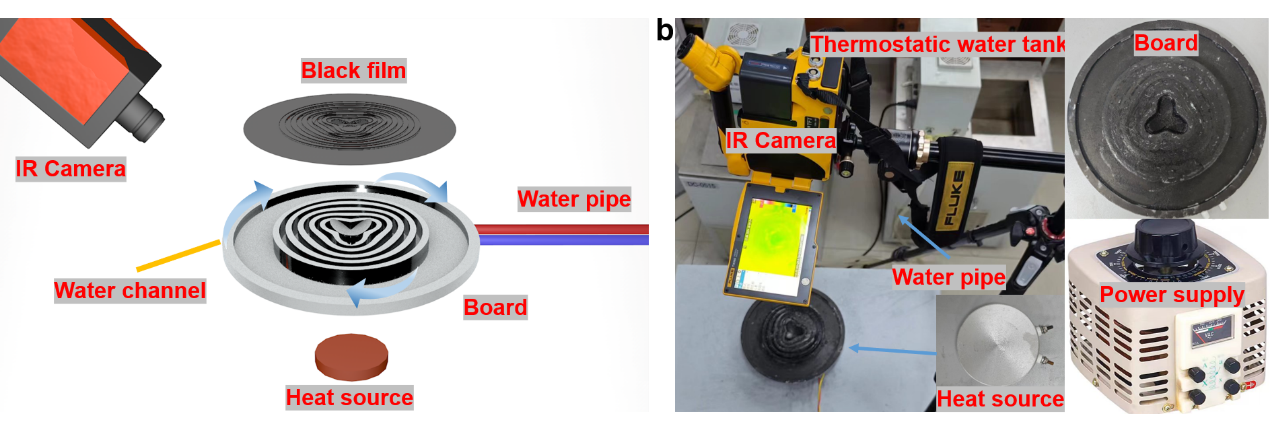


**Figure S22:** Actual experimental system diagram.


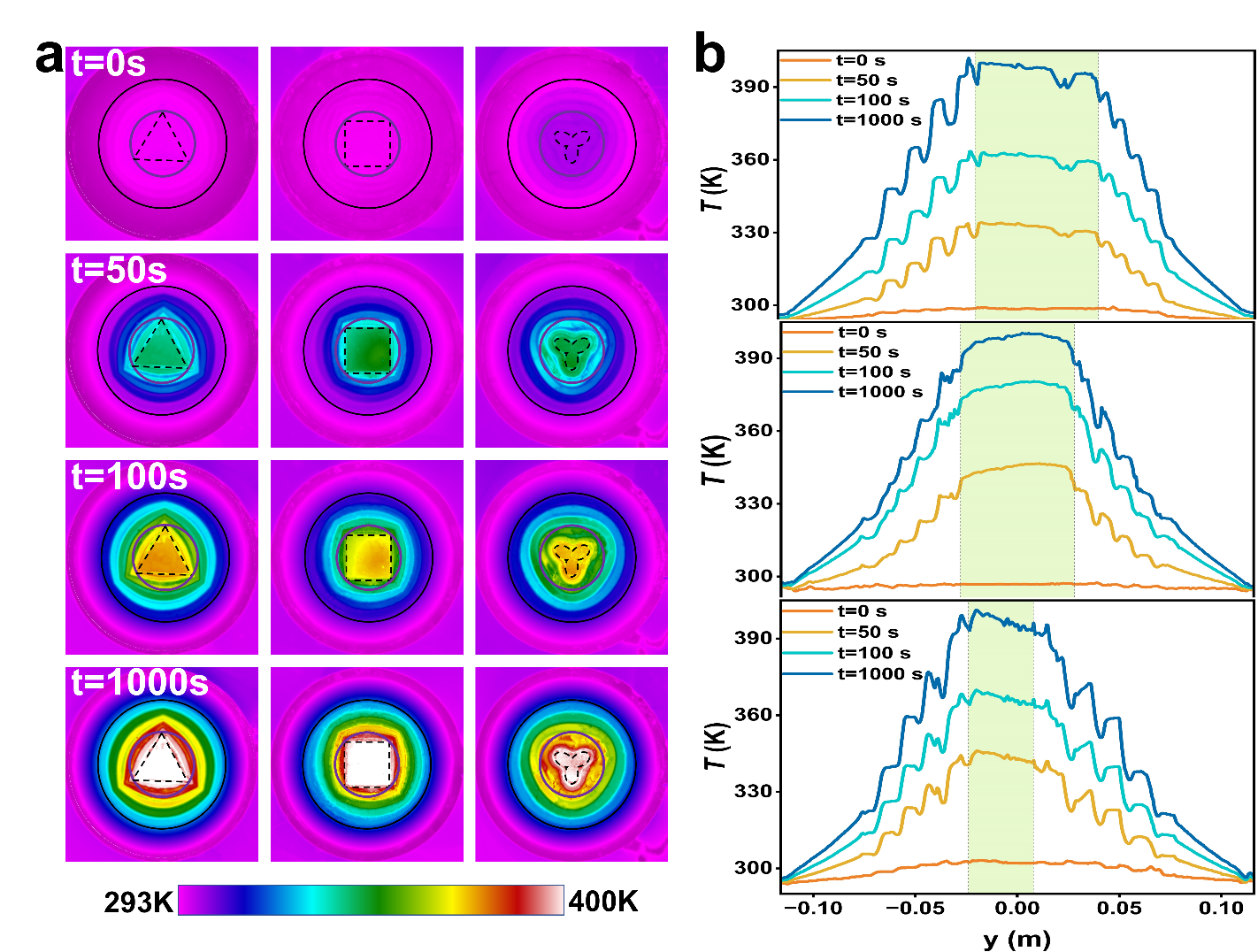


**Figure S23:** Experimental transient results for camouflaging a circular active source into triangular, square, and clover shapes: **a** Experimental transient results, and **b** Corresponding temperature profile along the central axis.


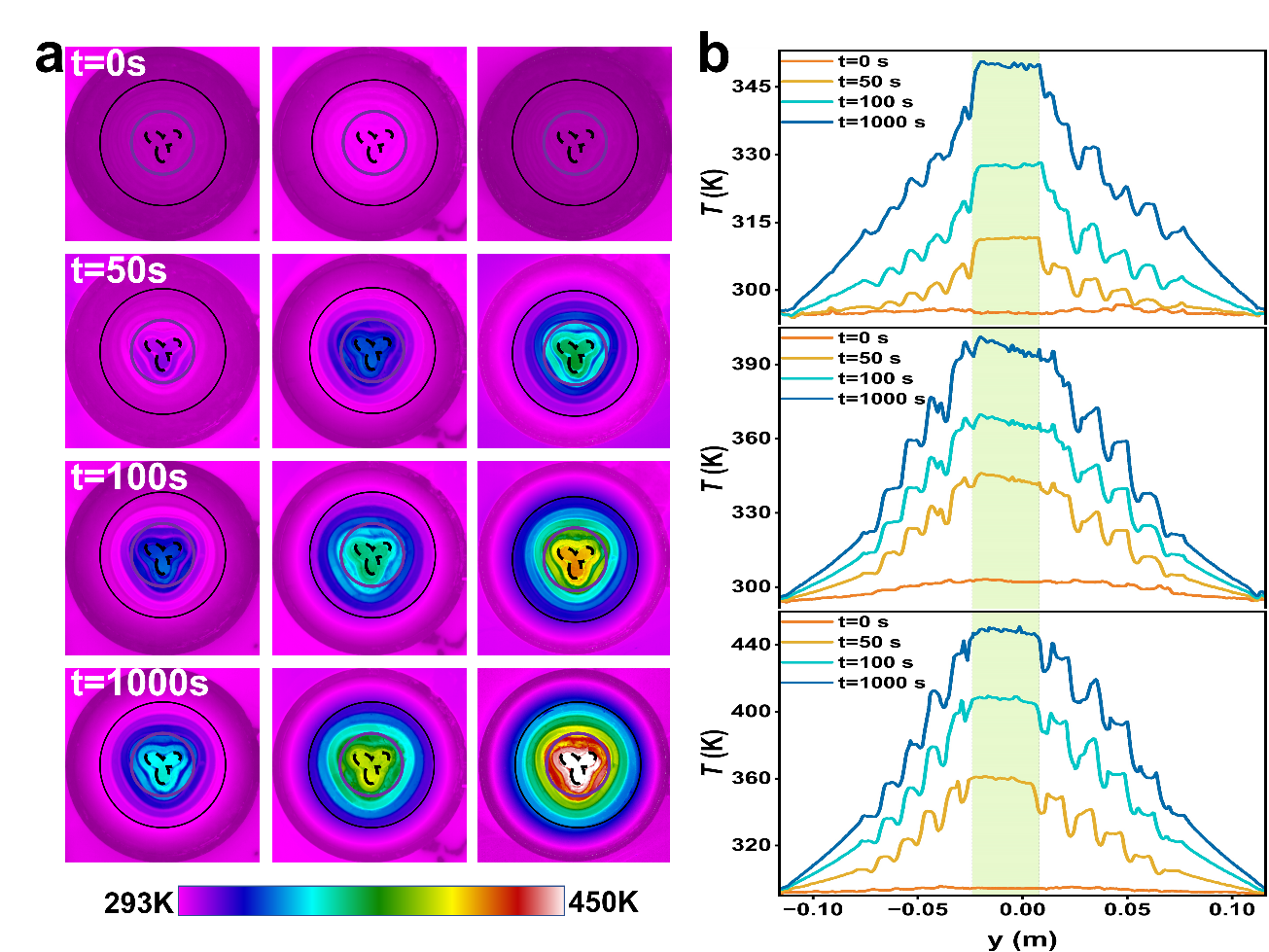


**Figure S24:** Experimental transient results for camouflaging a circular active source into a clover shape while regulating the active-source temperature to 350K, 400K, and 450K: **a** Experimental transient results, and **b** Corresponding temperature profile along the central axis.


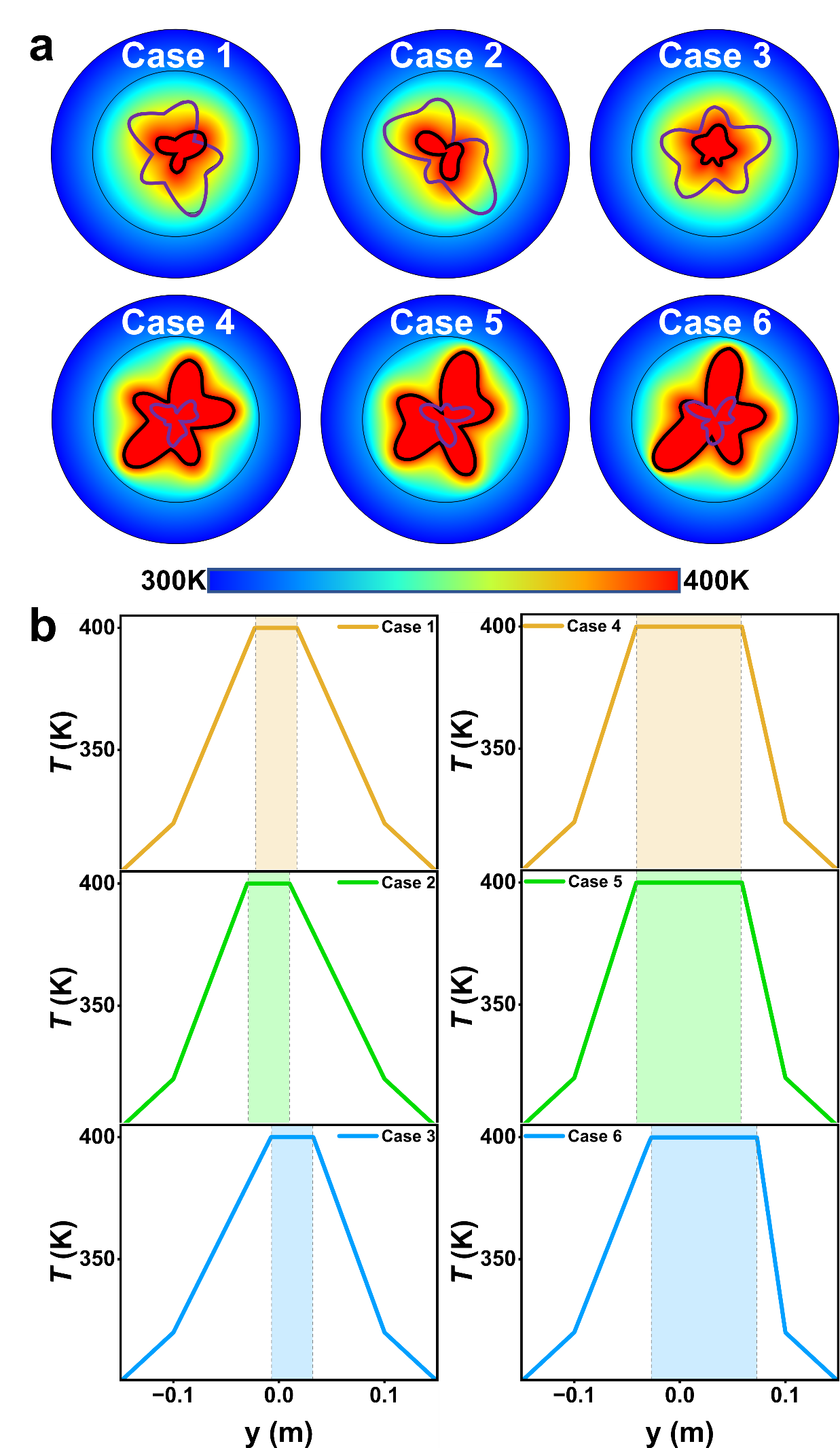


**Figure 25.** Examples of arbitrary complex active-source thermal camouflage. **a** The purple solid lines represent the actual active-source shape, while the black solid lines indicate the camouflaged active-source shape. **b** Temperature profile along the vertical central axis.


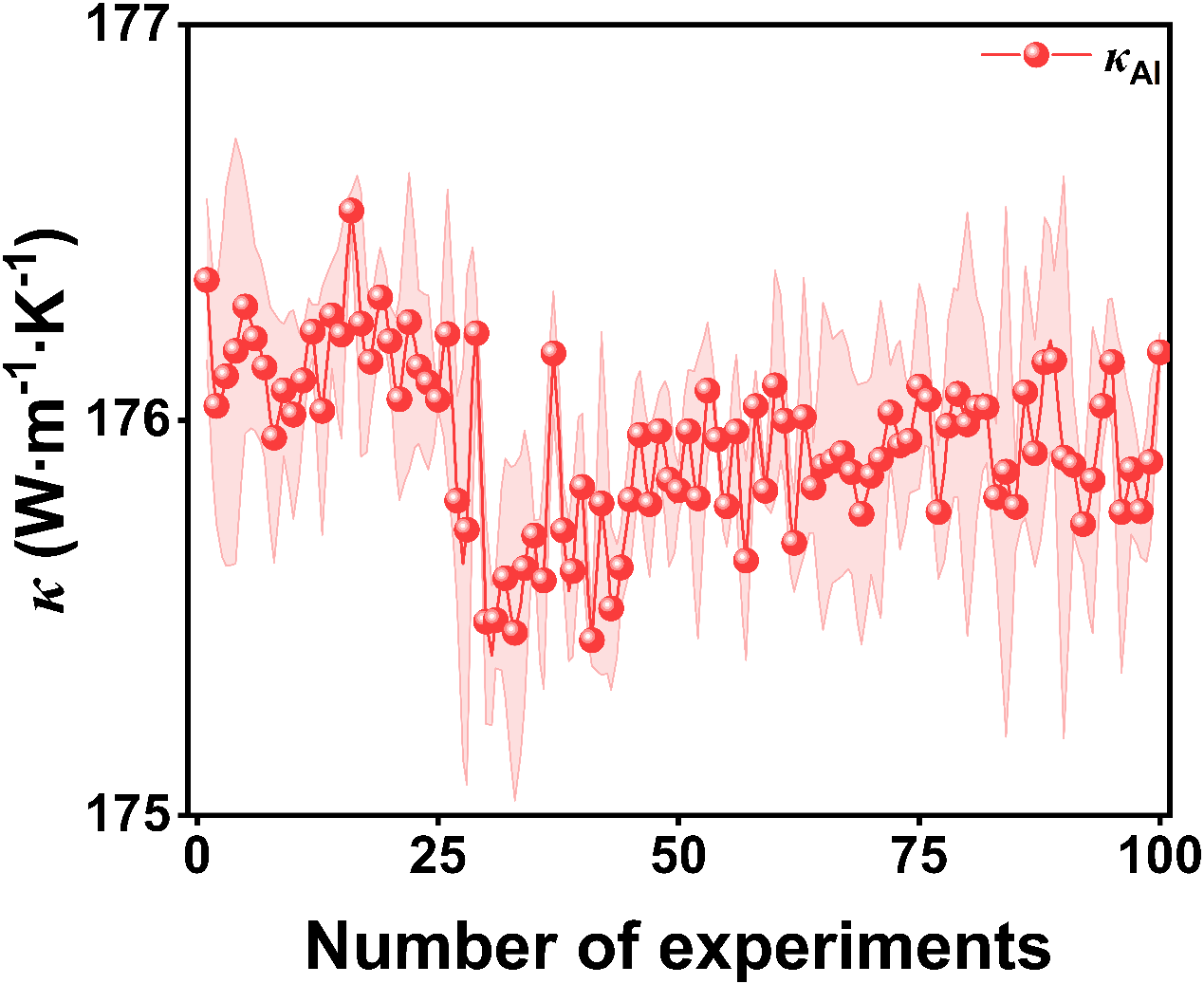


**Figure 26.** Thermal conductivity of the ASM over 100 heating cycles (from 293 K to 450 K).

**Table S1:** Heights of each layer in the structure of the experimental panel.

| Layers | Triangular  (400 K) | Square  (400 K) | Cloverleaf  (350 K) | Cloverleaf  (400 K) | Cloverleaf  (450 K) |
| --- | --- | --- | --- | --- | --- |
| 1 | 1.07 | 1.70 | 1.57 | 0.85 | 0.46 |
| 2 | 23.08 | 17.02 | 26.61 | 22.65 | 12.23 |
| 3 | 1.74 | 1.49 | 5.31 | 1.42 | 0.76 |
| 4 | 21.53 | 17.44 | 27.00 | 22.76 | 12.29 |
| 5 | 1.73 | 1.25 | 6.07 | 1.77 | 0.96 |
| 6 | 22.11 | 18.14 | 26.46 | 23.05 | 12.45 |
| 7 | 1.46 | 1.23 | 4.90 | 1.27 | 0.69 |
| 8 | 22.76 | 17.59 | 24.49 | 22.53 | 12.16 |
| 9 | 0.93 | 1.05 | 3.65 | 1.06 | 0.80 |
| 10 | 22.70 | 18.24 | 23.94 | 20.68 | 11.168 |
| Background | 2.50 | 2.50 | 2.50 | 2.50 | 2.50 |

Unit: mm

The camouflaged active-source region: $z=c+ar^{2}$, where c=0.00714 and a=8.

**References**

[1] R. Schittny, M. Kadic, S. Guenneau, M. Wegener, *Phys. Rev. Lett.* **2013**, *110*, 195901.

[2] J. Wang, G. Dai, J. Huang, *iScience* **2020**, *23*, 101637.

[3] J. B. Pendry, D. Schurig, D. R. Smith, *Science* **2006**, *312*, 1780.

[4] X. Xing, L. Wu, X. Dai, X. Tian, D. Li, *Int. J. Heat Mass Transf.* **2023**, *214*, 124437.

[5] U. Leonhardt, *Science* **2006**, *312*, 1777.

[6] F. Yang, Z. Zhang, L. Xu, Z. Liu, P. Jin, P. Zhuang, M. Lei, J. Liu, J.-H. Jiang, X. Ouyang, F. Marchesoni, J. Huang, *Rev. Mod. Phys.* **2024**, *96*, 015002.

[7] J. Guo, G. Xu, D. Tian, Z. Qu, C.-W. Qiu, *Adv. Mater.* **2022**, *34*, 2200329.

[8] R. Hu, S. Zhou, Y. Li, D.-Y. Lei, X. Luo, C.-W. Qiu, *Adv. Mater.* **2018**, *30*, 1707237.
